# Phylogenetic Evidence for Lateral Gene Transfer in the Intestine of Marine Iguanas

**DOI:** 10.1371/journal.pone.0010785

**Published:** 2010-05-24

**Authors:** David M. Nelson, Isaac K. O. Cann, Eric Altermann, Roderick I. Mackie

**Affiliations:** 1 Appalachian Laboratory, University of Maryland Center for Environmental Science, Frostburg, Maryland, United States of America; 2 Institute for Genomic Biology, University of Illinois, Urbana, Illinois, United States of America; 3 Department of Animal Sciences, University of Illinois, Urbana, Illinois, United States of America; 4 Department of Microbiology, University of Illinois, Urbana, Illinois, United States of America; 5 AgResearch Limited, Grasslands Research Centre, Palmerston North, New Zealand; Duke University Medical Center, United States of America

## Abstract

**Background:**

Lateral gene transfer (LGT) appears to promote genotypic and phenotypic variation in microbial communities in a range of environments, including the mammalian intestine. However, the extent and mechanisms of LGT in intestinal microbial communities of non-mammalian hosts remains poorly understood.

**Methodology/Principal Findings:**

We sequenced two fosmid inserts obtained from a genomic DNA library derived from an agar-degrading enrichment culture of marine iguana fecal material. The inserts harbored 16S rRNA genes that place the organism from which they originated within *Clostridium* cluster IV, a well documented group that habitats the mammalian intestinal tract. However, sequence analysis indicates that 52% of the protein-coding genes on the fosmids have top BLASTX hits to bacterial species that are not members of *Clostridium* cluster IV, and phylogenetic analysis suggests that at least 10 of 44 coding genes on the fosmids may have been transferred from *Clostridium* cluster XIVa to cluster IV. The fosmids encoded four transposase-encoding genes and an integrase-encoding gene, suggesting their involvement in LGT. In addition, several coding genes likely involved in sugar transport were probably acquired through LGT.

**Conclusion:**

Our phylogenetic evidence suggests that LGT may be common among phylogenetically distinct members of the phylum Firmicutes inhabiting the intestinal tract of marine iguanas.

## Introduction

*There is no other quarter of the world, where this order (reptiles), replaces herbivorous mammalia in so extraordinary a manner.* –Darwin [1]

During his visit to the Galápagos archipelago in 1835 Charles Darwin encountered several species of large herbivorous reptiles, and he made the intriguing observation that, with the exception of the Galápagos islands, there are few places on earth today where reptiles are the most abundant herbivores. The success of herbivorous reptiles on the Galápagos is, in part, related to their varied morphological, physiological, and behavioral adaptations [2], [3]. In addition, their effective utilization of plant material is aided by symbiotic relationships with intestinal microorganisms that hydrolyze and ferment the otherwise indigestible plant polymers [4], which is consistent with the known role of bacteria and protozoa in aiding digestion in herbivorous mammals [5] and insects [6].

**Figure 1:**
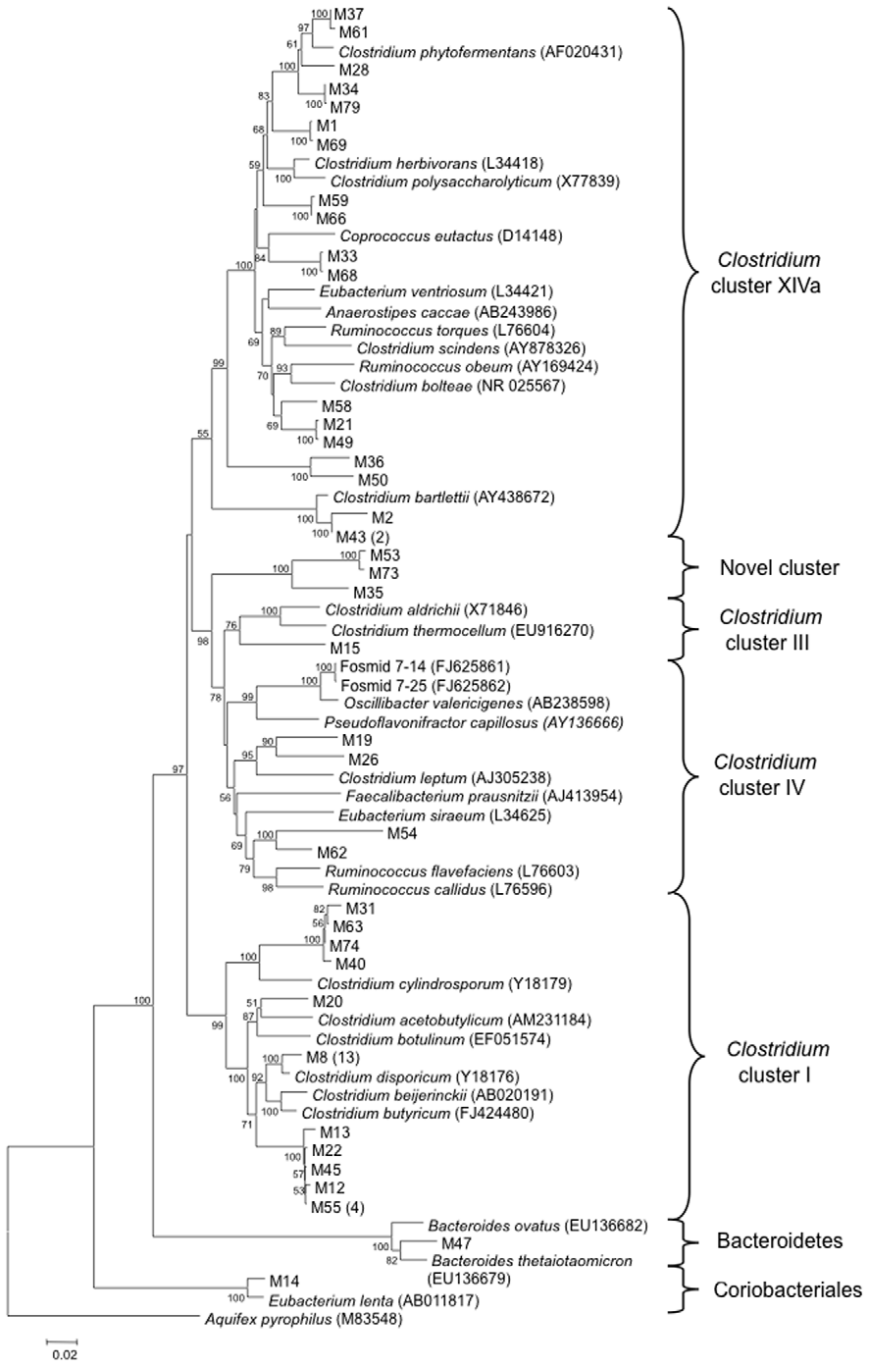
Phylogenetic relationships of 16S rRNA gene sequences among the fosmids, clone library, and *Clostridium* clusters. Clone library sequences start with “M.” The numbers in parentheses following some of the marine iguana sequences indicate the number of times that a particular sequence was obtained. Only representatives of the major *Clostridium* clusters, and limited representatives of the Bacteroidetes and Coriobacteriales, are shown. The tree was inferred using the neighbor joining approach. The numbers at the nodes represent bootstrap values. The bar represents 0.02 substitutions per nucleotide position. The outgroup is *Aquifex pyrophilus*.

In order to compete for resources and ultimately, to allow their host to survive and reproduce, intestinal microorganisms must also adapt. An emerging theme in genomic biology suggests that lateral gene transfer (LGT) is key for promoting genotypic and phenotypic variation in microorganisms [7], including those from intestinal environments [8], [9], [10], [11]. For example, Ricard et al. (2006) showed that ∼4% of genes in the genomes of ciliates common in the rumen were likely obtained from bacteria and archaea [11]. The majority of these genes were involved with carbohydrate catabolism, suggesting that their acquisition helped ciliates to successfully colonize and adapt to the rumen environment. Although the extent and control of LGT among microorganisms in the intestine of non-mammalian hosts, such as reptiles, remains unexplored, 16S rDNA clone libraries suggest that their gut bacterial communities differ in composition from those of herbivorous mammals. Firmicutes and specifically several phylogenetically defined *Clostridium* clusters (I, III, IV, and XIVa) are the predominant phyla in the intestine of marine iguanas (*Amblyrynchus cristatus*; Fig. 1) [4], land iguanas (*Conolophus* spp.), and giant tortoises (*Testudo elephantopus*) (Mackie, unpublished data). In contrast, herbivorous mammals also contain an abundance of diverse representatives of the phylum Bacteroidetes [12]. Thus if LGT is an important process in the intestine of herbivorous reptiles, it likely occurs among non-Bacteroidetes species.

The marine iguana, which is endemic to the Galápagos Islands, is unique among herbivorous reptiles because its diet consists solely of soft macrophytic algae. A previous study of Orkney sheep consuming a diet of seaweed indicated that such a diet selected for the proliferation of Candidatus *Oscillospira guilliermondii*, an uncultivated, but morphologically conspicuous, member of *Clostridium* cluster IV [13]. More recently, a closely related member of *Clostridium* cluster IV, *Oscillibacter valericigenes*, was isolated from the alimentary canal of clams that feed on marine plankton [14]. However, the ecology and evolution of *Oscillospira- and Oscillibacter*-like organisms remains poorly understood. To select for gut microbes capable of degrading agar (the primary component of the algal cell walls) we established an anaerobic enrichment culture from marine iguana fecal material using agar as a sole carbon source. We then created a fosmid library from the enrichment culture to obtain genomic information from organisms actively involved in agar-degradation in the hindgut of marine iguanas. We hypothesized that members of *Clostridium* cluster IV would be active in the culture, given their previously demonstrated abundance in the gastrointestinal tracts of marine animals. In the course of screening the library we discovered two fosmids with 16S rRNA gene sequence similar to those of *Oscillibacter valericigenes*, and an initial examination of the sequences using a BLAST-based approach suggested that some of the genes on the fosmids may have been subject to LGT. Here we report these sequence data and use a phylogenetic approach to assess the extent, probable direction, and mechanisms of LGT among these members of *Clostridium* cluster IV.

## Results and Discussion

The fosmid library that was constructed contained ∼2,000 clones. The fosmids selected for sequencing, named 7–14 and 7–25, were ∼35.3 and ∼29.5 kb in length, respectively. Each fosmid contained an RNA operon consisting of 16S rRNA, tRNA, and 23S rRNA genes (Table 1). The 16S and 23S rDNA tags on the fosmids were almost identical to each other (99.8% and 99.9% sequence similarity, respectively), and codon usage patterns on the fosmids were significantly correlated (r = 0.70, p<0.001), which indicates that the fosmids likely derive from the same species. Phylogenetic analysis, based on 16S rDNA, indicated that the fosmids were derived from members of *Clostridium* cluster IV with ∼97.3% 16S rDNA similarity to their nearest cultivated relative, *Oscillibacter valericigenes* (Fig. 1). The nearest cultivated relative with a complete genome sequence available in a public database is *Bacteroides capillosus*, which has now been reclassified as *Pseudoflavonifractor capillosus*, a member of *Clostridium* cluster IV, based on biochemical properties, DNA G+C content, DNA-DNA hybridization and phylogenetic position [15].

**Table 1:** 

*Fosmid name*	*CDS* a	*Nucleotide range*	*Transcription direction*	*% GC content*	*Predicted function* b	*LGT? (probable direction of transfer)* c	*BLASTX analysis* d			
							*Name of top hit*	*Accession number*	*E value*	*% Nucleotide identity*
7–14	1	18–920	+	53.0	**Transposase for insertion sequence element ISRM5**	No	***Pseudoflavonifractor capillosus***	ZP_02038860	2.00E−136	92
7–14	2	1459–1929	−	35.6	Teicoplanin resistance protein vanZ	Novel	*Geobacillus thermodenitrificans*	YP_001124564	7.00E−06	31
7–14	3	2346–3584	−	50.8	**Putative transposase**	**Yes, from ** ***Clostridium*** **XIVa to IV**	*Coprococcus eutactus*	ZP_02205788	2.00E−167	69
7–14	4	4214–5032	−	43.8	Hypothetical protein	Novel	*Anaerostipes caccae*	ZP_02418355	1.00E−03	39
7–14	5	6252–6962	−	51.9	Outer membrane lipoprotein-sorting protein	Novel	*Caldicellulosiruptor saccharolyticus*	YP_001179297	8.00E−06	31
7–14	6	6959–7480	−	47.3	RNA polymerase sigma-54 factor rpoN	Unresolved	*Alkaliphilus oremlandii*	YP_001512203	1.00E−28	40
7–14	7	7745–7820		63.2	tRNA-Pro (TGG)	Na				
7–14	8	8265–10187	−	57.9	Large exoproteins involved in heme utilization or adhesion	Unresolved	*Herpetosiphon aurantiacus*	YP_001545021	2.00E−58	35
7–14	9	10188–10862	−	55.4	Hypothetical protein	Unresolved	***Anaerotruncus colihominis***	ZP_02440985	3.00E−38	56
7–14	10	10855–11580	−	54.4	Hypothetical protein	**Yes, direction unresolved**	*Desulfitobacterium hafniense*	YP_517449	7.00E−67	56
7–14	11	11732–12193	−	52.8	Iron-sulfur cluster regulator IscR	Unresolved (No)	***Anaerotruncus colihominis***	ZP_02443971	2.00E−44	65
7–14	12	12428–13360	+	59.1	Cysteine synthase	Unresolved	***Faecalibacterium prausnitzii***	ZP_02090920	4.00E−123	83
7–14	13	13706–13840	−	46.7	Sodium/glutamate symporter	Unresolved	***Eubacterium siraeum***	ZP_02423775	5.00E−12	80
7–14	14	13795–14877	−	56.7	Sodium/glutamate symporter	Unresolved	***Eubacterium siraeum***	ZP_02423775	7.00E−134	73
7–14	15	14895–15938	−	61.3	Immunogenic protein	Unresolved	*Coprococcus eutactus*	ZP_02205701	6.00E−61	44
7–14	16	16214–17023	−	57.9	8-oxoguanine-DNA-glycosylase	**Yes, from ** ***Clostridium*** ** XIVa to IV**	*Ruminococcus torques*	ZP_01966859	9.00E−55	43
7–14	17	17025–18026	−	57.6	L-asparaginase	Unresolved	*Clostridium bolteae*	ZP_02087774	7.00E−103	54
7–14	18	18036–19133	−	62.6	Exonuclease SbcD	No	***Pseudoflavonifractor capillosus***	ZP_02034908	3.00E−98	55
7–14	19	19130–19684	−	56.8	EBSC protein	Unresolved	***Clostridium leptum***	ZP_02078782	2.00E−54	68
7–14	20	19651–20424	−	55.9	Phosphoesterase family protein	No	***Pseudoflavonifractor capillosus***	ZP_02034909	4.00E−87	61
7–14	21	21133–21414	−	50.7	Hypothetical protein	Unresolved	***Eubacterium siraeum***	ZP_02423392	2.00E−08	35
7–14	22	22289–23032	+	54.6	**Transposase for insertion sequence element ISRM5**	No	***Pseudoflavonifractor capillosus***	ZP_02038860	1.00E−110	87
7–14	23	23434–23508		58.7	tRNA-Glu (CTC)	Na				
7–14	24	23684–23759		52.6	tRNA-Lys (CTT)	Na				
7–14	25	23856–26709		52.5	23S rRNA gene	Na				
7–14	26	27033–27109		63.6	tRNA-Ile (GAT)	Na				
7–14	27	27121–27196		53.9	tRNA-Ala (TGC)	Na				
7–14	28	27324–28847		53.5	16S rRNA gene	Na				
7–14	29	29448–30365	−	57.4	Germination and sporulation	Yes, from XIVa to IV (No)	***Pseudoflavonifractor capillosus***	ZP_02038021	1.00E−25	38
7–14	30	30362–31768	−	60.5	Osmosensitive K+ channel histidine kinase kdpD	No	***Pseudoflavonifractor capillosus***	ZP_02038022	1.00E−120	52
7–14	31	31788–32465	−	55.8	Two-component response regulator SA14-24	No	***Pseudoflavonifractor capillosus***	ZP_02036840	7.00E−86	76
7–14	32	32486–32854	−	55.3	Late competence protein comEA, DNA receptor	Unresolved	Cand. *Desulforudis audaxviator*	YP_001718193	3.00E−15	58
7–14	33	33191–34558	+	57.1	D-alanyl-D-alanine carboxypeptidase	No	***Pseudoflavonifractor capillosus***	ZP_02036838	3.00E−7	47
7–14	34	34607–35155	−	59.7	Nitroreductase family protein	Unresolved (Yes, from XIVa to IV)	*Clostridium kluyveri*	YP_001393744	9.00E−36	50
7–25	1	1–794	+	51.5	**Integrase**	**Yes, from ** ***Clostridium*** ** XIVa to IV**	*Clostridium bolteae*	ZP_02083674	6.00E−90	62
7–25	2	910–2469	−	57.8	GMP synthase [glutamine-hydrolyzing]	No	***Pseudoflavonifractor capillosus***	ZP_02035344	0.00E+00	82
7–25	3	2447–2962	−	56.2	Xanthine phosphoribosyltransferase	Unresolved	*Coprococcus eutactus*	ZP_02207218	3.00E−43	54
7–25	4	3418–4239	−	60.5	Nucleotide-binding protein	No	***Eubacterium siraeum***	ZP_02421312	4.00E−91	64
7–25	5	4308–6572	−	61.3	Chromosome partition protein smc	No	***Pseudoflavonifractor capillosus***	ZP_02034907	6.00E−103	35
7–25	6	7160–8682		53.6	16S rRNA gene	Na				
7–25	7	8810–8885		55.3	tRNA-Ala (TGC)	Na				
7–25	8	8897–8973		64.9	tRNA-Ile (GAT)	Na				
7–25	9	9297–12150		52.5	23S rRNA gene	Na				
7–25	10	12247–12322		53.9	tRNA-Lys (CTT)	Na				
7–25	11	12498–12572		58.7	tRNA-Glu (CTC)	Na				
7–25	12	12864–12939		57.9	tRNA-Asn (GTT)	Na				
7–25	13	12994–13070		61.0	tRNA-Met (CAT)	Na				
7–25	14	13108–13183		59.2	tRNA-Trp (CCA)	Na				
7–25	15	13246–13322		62.3	tRNA-Asp (GTC)	Na				
7–25	16	13328–13403		59.2	tRNA-Thr (GGT)	Na				
7–25	17	13613–14674	+	54.3	**Transposase for insertion sequence element ISRM5**	No	***Pseudoflavonifractor capillosus***	ZP_02038860	4.00E−131	89
7–25	18	15027–15791	+	40.4	Unknown	Unresolved (No)	*Bacteroides thetaiotaomicron*	NP_810500	1.00E−117	78
7–25	19	15913–17193	+	55.2	Hypothetical protein	Unresolved	***Eubacterium siraeum***	ZP_02421339	4.00E−86	44
7–25	20	17144–18088	+	56.9	Hypothetical protein	Unresolved	*Eubacterium ventriosum*	ZP_02027484	5.00E−66	43
7–25	21	18370–19662	+	58.0	Putative stomatin/prohibitin-family membrane protease subunit	**Yes, direction unresolved**	*Clostridium acetobutylicum*	NP_349972	2.00E−92	48
7–25	22	19560–20867	+	57.0	Protein RtcB	No	***Pseudoflavonifractor capillosus***	ZP_02038919	2.00E−154	69
7–25	23	20864–21208	+	55.1	Predicted nucleotidyltransferase	Unresolved	*Escherichia coli*	ZP_03048733	5.00E−16	59
7–25	24	22686–23951	+	53.7	Hypothetical lipoprotein	**Yes, from ** ***Clostridium*** ** XIVa to IV**	*Clostridium bolteae*	ZP_02087827	0.00E+00	82
7–25	25	24010–25620	+	48.6	ABC-type sugar transport system, ATP-binding protein	**Yes, from ** ***Clostridium*** ** XIVa to IV**	*Clostridium bolteae*	ZP_02087828	0.00E+00	89
7–25	26	25634–26713	+	54.8	ABC transporter integral membrane protein	**Yes, from ** ***Clostridium*** ** XIVa to IV**	*Clostridium bolteae*	ZP_02087829	2.00E−164	88
7–25	27	26710–27834	+	54.3	ABC transporter integral membrane protein	**Yes, from ** ***Clostridium*** ** XIVa to IV**	*Clostridium bolteae*	ZP_02087830	7.00E−167	84
7–25	28	27831–28319	+	54.6	Hypothetical protein	**Yes, direction unresolved**	*Clostridium bolteae*	ZP_02087831	6.00E−47	70

Na, not applicable.

a CDS number from 5′ to 3′ on cloned insert.

b The predicted function of each gene was determined as desribed in the text. Predicted mobile elements are in bold.

c The occurrence and direction of LGT was determined using phylogenetic analysis, as described in the text. Genes that likely underwent LGT are in bold.

For CDSs in which the assessment of LGT differed between the neighbor joining and maximum likelihood based approaches the neighbor joining assessment is listed first and the maximum likelihood assessment is listed in parentheses.

d As compared to the protein sequence database in GenBank. Top BLASTX hits that are members of *Clostridium* cluster IV are in bold.

*Oscillibacter* 16S rRNA gene sequences were not recovered from the small clone library created from genomic DNA extracted from marine iguana fecal material. Nevertheless, we successfully amplified 16S rDNA sequences using primers unique to the fosmids from 4/5 fecal samples from 5 different marine iguanas (Fig. S1), confirming the presence of the bacteria that the fosmids represent in the original fecal material. To ensure that the primers amplified the 16S rRNA gene sequences identified in the fosmid sequences, we extracted DNA and then cloned and sequenced 16S rDNA from one sample (sample 24). As anticipated, the top BLASTN hits of two clones that were sequenced (GenBank accession numbers GQ243725 and GQ243726) were fosmids 7–14 and 7–25. These results confirm that the organisms that the fosmids represent are present in marine iguana fecal material and are commonly found in the intestinal tracts of marine iguanas (Fig. S1).

**Figure 2:**
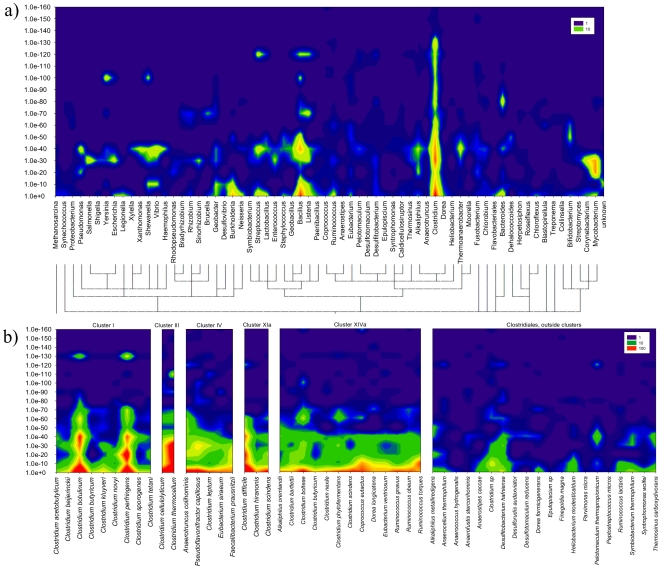
BLASTP result distribution across fosmids 7–14 and 7–25. a) The X-axis indicates genera with at least 10 BLASTP hits throughout the ORFeome of the analyzed fosmids. Using a previously published approach [33] the organism distribution on a genus level was identified for each coding gene, e-values were grouped into ranges, and threshold levels were defined for minimum overall frequency. Genera are phylogenetically sorted. The Y-axis indicates respective e-value ranges. The frequency of hits for each genus in each e-value range (log scale) is shown by color coding and corresponding values are indicated in the figure. All BLASTP hits per genus per ORF were accepted. b) Same as a), except that custom databases of species from phylogenetically defined *Clostridium* clusters were used.

Consistent with the fact that their 16S rRNA genes indicate that the fosmids are members of *Clostridium* cluster IV (Fig. 1), the most dominant flare in a BLAST Heat Map of the coding genes was observed compared to the genus *Clostridium*. However, zones of relatively conserved sequences (e-values<1e-80) are also evident in comparison with other genera (Fig. 2a), and a concatenated BLAST Heat Map, created using custom databases of *Clostridium* clusters, displays flares with members outside of *Clostridium* cluster IV (Fig. 2b). In addition, over half (52%) of the protein-coding genes on the fosmids have top BLASTX hits to bacteria that are not members of *Clostridium* cluster IV (Table 1). These results contrast with phylogenetic relationships based on 16S rRNA gene sequences, and they suggest potential exchange of genetic material between phylogenetically distinct groups of bacteria.

To more rigorously assess which genes may have been subject to LGT we used phylogenetic analysis, in conjunction with parsimony analysis [9]. Assessments of LGT using neighbor-joining (NJ) and approximately maximum-likelihood (ML) trees were congruent for all but four of the coding genes. In two cases (CDS 11 on fosmid 7–14 and CDS 18 on fosmid 7–25, Figs. S2 and S3, respectively) the NJ trees could not resolve the occurrence of LGT and the ML trees suggested that LGT did not occur (Table 1). For one coding gene (CDS 29 on fosmid 7–14, Fig. S2) the NJ tree suggested LGT, whereas the ML tree indicated no LGT (Table 1). Bootstrap support for the NJ tree was low (61) and thus this gene was unlikely to have experienced recent LGT. For one coding gene (CDS 34 on fosmid 7–14, Fig. 2) LGT was unresolved with the NJ approach, whereas the ML approach suggested the occurrence of LGT. Thus we conservatively estimate that at least 10 of 44 coding genes on the fosmids (Table 1, Figs. S2 and S3) had been subject to LGT, which confirms that LGT is an important process in the evolution of intestinal microorganisms in marine iguanas. Although the precise proportion of genes subject to LGT on fosmids 7–14 and 7–25 may differ from the extent of LGT in the genome from which the fosmids derive, these results nevertheless indicate the occurrence of LGT. For all cases in which the direction of LGT could be resolved (i.e. 7 of 10 cases), the transfers likely occurred from *Clostridium* cluster XIVa to cluster IV. Representatives of *Clostridium* clusters XIVa and IV are predominated by intestinal bacteria and are common in marine iguana fecal material (Fig. 1). Thus our finding of the acquisition of genetic material by organisms in *Clostridium* cluster IV from those in cluster XIVa is reasonable.

The presumed split of *Clostridium* clusters XIVa and IV cannot explain the occurrence of genes from *Clostridium* cluster XIVa within the fosmids, because the phylogenetic results indicate that the gene transfers likely occurred after these organisms diverged. When phylogenetic analysis based on the NJ and ML trees indicated that a particular gene was likely not subject to LGT, the top BLASTX hit was from *Clostridium* cluster IV, whereas when phylogenetic analysis indicated the gene was subject to LGT the top BLAST hit was not from *Clostridium* cluster IV (Table 1). It is possible that some coding genes for which the occurrence of LGT could not be resolved using phylogenetic analysis were also subject to LGT as indicted by their lack of top BLASTX hits to members of *Clostridium* cluster IV (Table 1). These results suggest an occurrence of the exchange of genetic material between phylogenetically distinct groups of intestinal microbes in herbivorous reptiles, consistent with recent evidence for the occurrence of LGT among microorganisms in the intestine of mammalian herbivores [9], [11], [16], as well as in marine water and sediment [7], [17].

In addition, our sequence analysis indicated a total of four transposase-encoding genes and an integrase-encoding gene on the fosmids, which provides circumstantial evidence of a potential mechanism for facilitation of LGT (Table 1). Three of the transposase-encoding genes are native to *Clostridium* cluster IV (not subject to LGT), and two of these appear to have interrupted the RNA operons on their respective fosmids. One transposase-encoding gene and an integrase-encoding gene appear to have originated from *Clostridium* cluster XIVa, suggesting their potential role in transferring genes specific to *Clostridium* cluster XIVa into the genomes of members of cluster IV.

Together, these results indicate the exchange of genetic material between phylogenetically distinct Clostridia found within the intestine of the marine iguana that are potentially involved in agar degradation and are the subject of ongoing research. Some of the transferred genes may have functions particularly valuable for enabling bacteria to colonize and survive in the marine iguana intestine. For example, a primary role of bacteria in the intestine of the marine iguana is to degrade algal polysaccharides (e.g. agar and agaropectin), found in the cell walls of macrophytic algae, into simple sugars. These sugars may then be transported into bacterial cells. The acquisition of new types of transporters through LGT may increase the types of sugars from which microorganisms may obtain energy, and *Oscillospira* has been shown to rapidly associate with freshly ingested forage [18]. Although the precise timing of the LGT events revealed on the fosmids is unclear, there is evidence that some transfers may have occurred recently in evolutionary history. For example, the ABC transporters on fosmid 7–25 have synteny with, and high nucleotide-level sequence similarity to, sequences found in *Clostridium bolteae* (Table 1 and Fig. 1), suggesting little divergence and a relatively recent transfer event. Indeed, a recent review concludes that transfers of complex protein-encoding genes, many of which are located on operons and gene clusters, could be very common [19].

Conversely, other genes subject to LGT have less nucleotide-level sequence similarity, suggesting more ancient transfers (Table 1). Thus LGT appears to be a means for microorganisms in the intestine of herbivorous reptiles to acquire new functions and adapt to changing environmental conditions. These results, combined with other recent studies, indicate that the high microbial density and diversity of the rumen and other intestinal ecosystems create an environment conducive to LGT [8].

## Materials and Methods

Fresh fecal material from 5 individual marine iguanas was collected and stored at −20°C. All procedures were non-invasive and conducted in accordance with guidelines from the American Society of Icthyologists and Herpetologists, approved by the Charles Darwin Research Station and covered under University of Illinois Urbana-Champaign LACAC #03041 and Princeton University IACUC #1428. To assess overall bacterial community composition in feces a 16S rDNA clone library was created from pooled genomic DNA. DNA was extracted using the UltraClean Soil DNA kit (MO BIO Laboratories, Carlsbad, CA). The primers used for PCR amplification of DNA from the pooled fecal samples were 27f and 1525r [20]. Amplicons were directly cloned into the PCRII-TOPO cloning vector (Invitrogen, Carlsbad, CA), and recombinant plasmids were extracted using the Wizard® Plus Minipreps DNA Purification System (Promega, Madison, WI). Sequencing was performed by the W.M. Keck Center for Comparative and Functional Genomics at the University of Illinois Urbana-Champaign.

An enrichment culture from the fecal material was created using agar as the sole carbon source in anaerobic medium [21]. The culture actively degraded agar as evidenced by rapid liquefaction. However, repeated attempts to isolate pure cultures of organisms capable of agar-degradation failed. A fosmid library was created from the fecal material using previously described methods [22]. The fosmid library was screened using PCR for those harboring inserts with a phylogenetic tag, the 16S rRNA gene, from *Clostridium* cluster IV [23], a heterogeneous group that includes non-clostridial species and is abundant in the intestine of the marine iguana (Fig. 1). The complete sequences of two of these fosmids, named 7–14 and 7–25, were obtained using Sanger sequencing and a “primer walking” approach. The sequences of fosmids 7–14 and 7–25 (GenBank accession numbers FJ625861 and FJ625862, respectively) were analyzed in the SEED Annotation Engine in RAST (http://rast.nmpdr.org/, Version 2.0) in order to identify genes and determine their predicted function [24]. Putative tRNA genes were folded using tRNA-scan [25] to confirm their identity.

The 16S rRNA gene sequences from the fosmids, the clone library, and representatives of the major *Clostridium* clusters, and limited representatives of the Bacteroidetes and Coriobacteriales were aligned using CLUSTAL W [26]. Evolutionary distances were calculated using the method of Kimura [27], and phylogenetic trees were inferred using the NJ [28] and maximum parsimony [29] methods in the MEGA 3.1 software package [30]. An approximately maximum-likelihood (ML) phylogenetic tree was also inferred using FastTree 2.1.2 [31]. All trees were concordant with each other. We also aligned the sequences using a core set of 16S rRNA gene sequences (i.e. http://greengenes.lbl.gov) and the resulting phylogenetic trees were concordant with those derived from sequences that were aligned using CLUSTAL W. To verify the presence of the 16S rDNA sequences of fosmids 7–14 and 7–25 in marine iguana fecal samples we randomly selected and extracted genomic DNA from 5 other samples of fresh fecal material. Primers unique to the 16S rDNA sequence of the fosmids (99f, 5′-AATGTTTAGTGGCGGACTGG-3′, and 1503r, 5′-ACCTTCCGATACGGCTACCT-3′) were designed and used to amplify the genomic DNA.

NJ and approximately ML phylogenetic trees of amino acid sequences from fosmids 7–14 and 7–25 were used to assess LGT, as described by Xu et al. [9]. Briefly, genes were marked as “novel” if they had e-values>10^−6^. If e-values were<10^−6^ we started at the query sequence and then stepped back in the tree until a bootstrap-supported node (>60) that contained sequences from a different species was found. If the node had decedents only from *Clostridium* cluster IV the gene was marked as “no LGT.” If the node had decedents from within and outside of *Clostridium* cluster IV the gene was marked as “unresolved.” If the node had decedents only from outside of *Clostridium* cluster IV the gene was marked as laterally transferred. Genes marked as laterally transferred were then subject to Fitch parsimony analysis [32] in order to determine the ancestral state of each node and the probable direction of transfer, when possible.

## Supporting Information

Figure S1 PCR assessment of the presence of fosmid 7–14 and 7–25 16S rDNA sequences in marine iguana fecal samples from five different marine iguanas (named 24, 10, 18, 26, and 19). Arrows point to the 1.4 and 1.5 kb markers, between which is the expected PCR product size. Lanes 1 and 10 are molecular weight ladders (M). Lanes 2–6 represent the samples. A faint band of the expected size is present in sample 10, whereas no bad is visible in sample 18. Lanes 7–8 are positive controls (DNA from fosmids 7–14 and 7–25), and lane 9 is negative control (−).
(0.15 MB PDF)

Figure S2 Neighbor-joining phylogenetic trees of amino acid sequences from fosmid 7–14 that were used to assess LGT, as described in the text. Sequences in bold represent those from *Clostridium* cluster IV. For CDS 11, 29, and 34 the conclusion of LGT based upon the neighbor-joining trees differed from that based upon maximum likelihood trees (as listed in Table 1). Thus for these CDSs we also show the maximum likelihood trees.
(4.49 MB PDF)

Figure S3 Neighbor-joining phylogenetic trees of amino acid sequences from fosmid 7–25 that were used to assess LGT, as described in the text. Sequences in bold represent those from *Clostridium* cluster IV. For CDS 18 the conclusion of LGT based upon the neighbor-joining tree differed from that based upon the maximum likelihood tree (as listed in Table 1). Thus for this CDS we also show the maximum likelihood tree.
(3.02 MB PDF)
