# Supplementary figures and images for: Phylogenetic Evidence for Lateral Gene Transfer in the Intestine of Marine Iguanas
Source: PLoS One. 2010 May 24;5(5):e10785. doi: 10.1371/journal.pone.0010785 (PMC2875401; doi:10.1371/journal.pone.0010785)

Figure S1

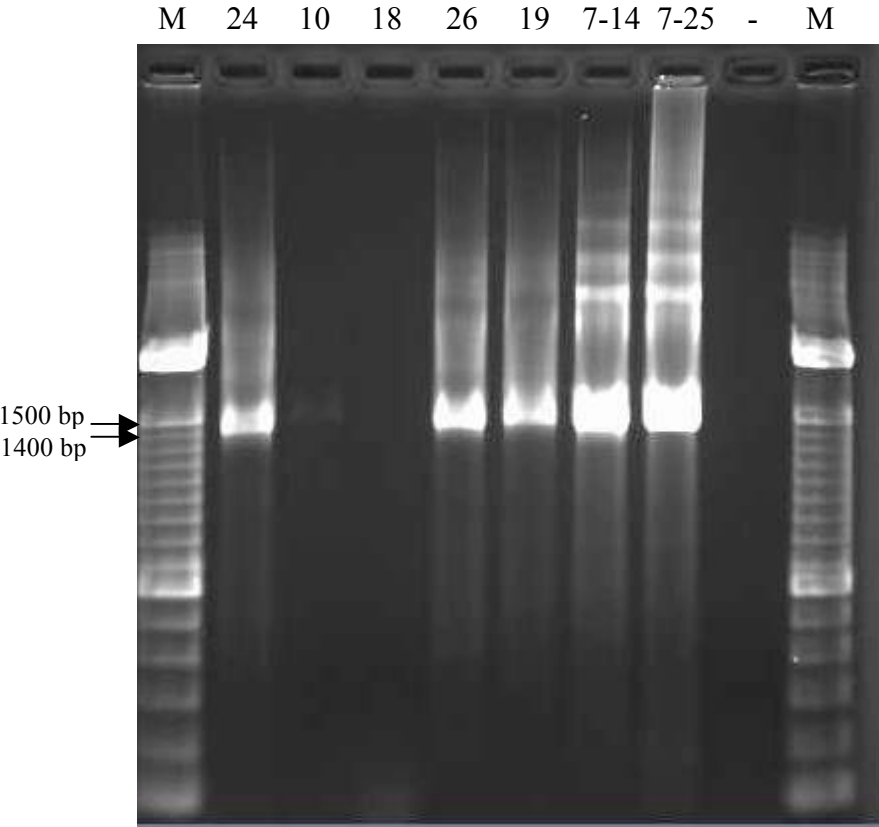

Supplement: Figure S1 — PCR assessment of the presence of fosmid 7–14 and 7–25 16S rDNA sequences in marine iguana fecal samples from five different marine iguanas (named 24, 10, 18, 26, and 19). Arrows point to the 1.4 and 1.5 kb markers, between which is the expected PCR product size. Lanes 1 and 10 are molecular weight ladders (M). Lanes 2–6 represent the samples. A faint band of the expected size is present in sample 10, whereas no bad is visible in sample 18. Lanes 7–8 are positive controls (DNA from fosmids 7–14 and 7–25), and lane 9 is negative control (−). (0.15 MB PDF) [file pone.0010785.s001.pdf]
