# Supplementary material for: Phylogenetic Evidence for Lateral Gene Transfer in the Intestine of Marine Iguanas
Source: PLoS One. 2010 May 24;5(5):e10785. doi: 10.1371/journal.pone.0010785 (PMC2875401; doi:10.1371/journal.pone.0010785)

Figure S2

a) CDS1

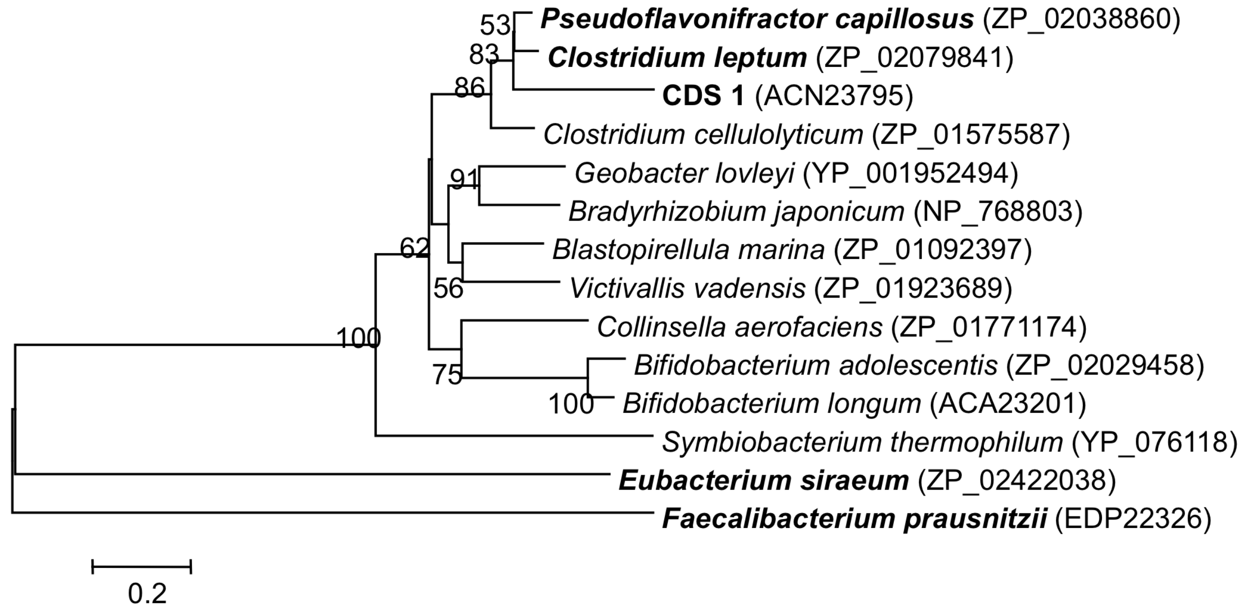

b) CDS 3

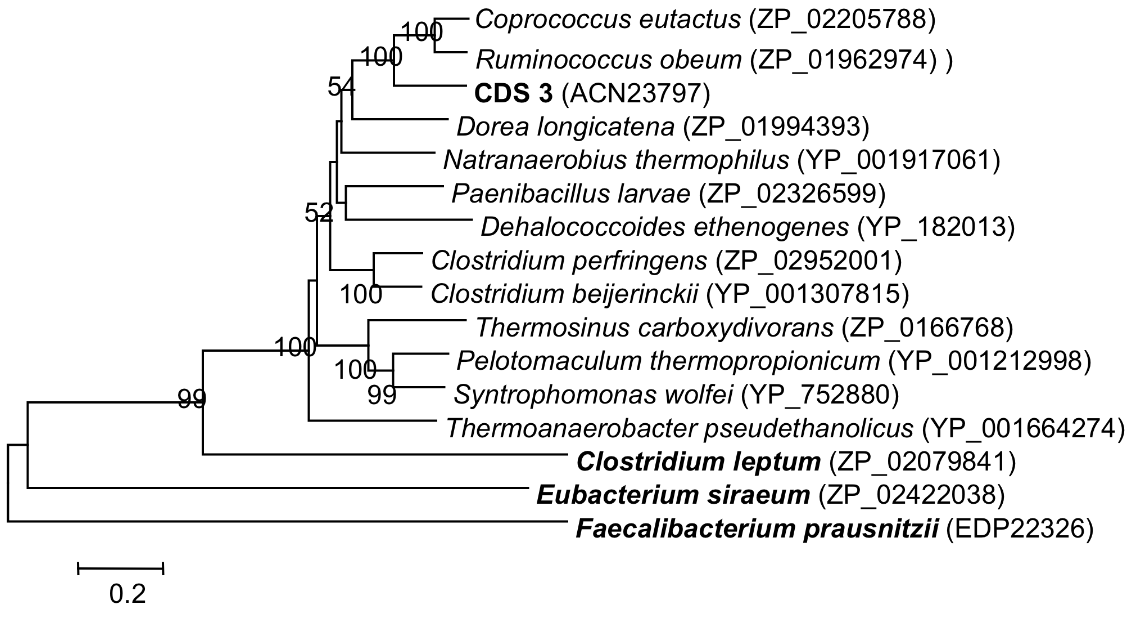

c) CDS 6

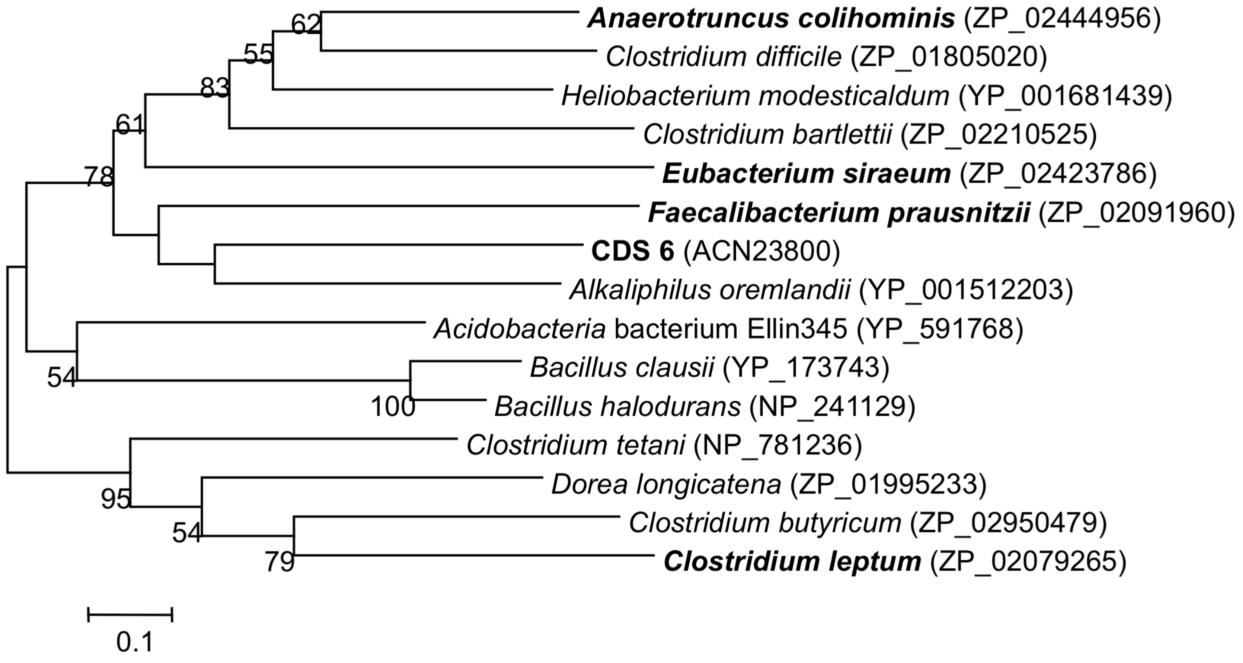

62
55
83
61
78
54
100
95
54
79
Anaerotruncus colihominis (ZP_02444956)
Clostridium difficile (ZP_01805020)
Heliobacterium modesticaldum (YP_001681439)
Clostridium bartlettii (ZP_02210525)
Eubacterium siraeum (ZP_02423786)
Faecalibacterium prausnitzii (ZP_02091960)
CDS 6 (ACN23800)
Alkaliphilus oremlandii (YP_001512203)
Acidobacteria bacterium Ellin345 (YP_591768)
Bacillus clausii (YP_173743)
Bacillus halodurans (NP_241129)
Clostridium tetani (NP_781236)
Dorea longicatena (ZP_01995233)
Clostridium butyricum (ZP_02950479)
Clostridium leptum (ZP_02079265)
0.1

d) CDS 8

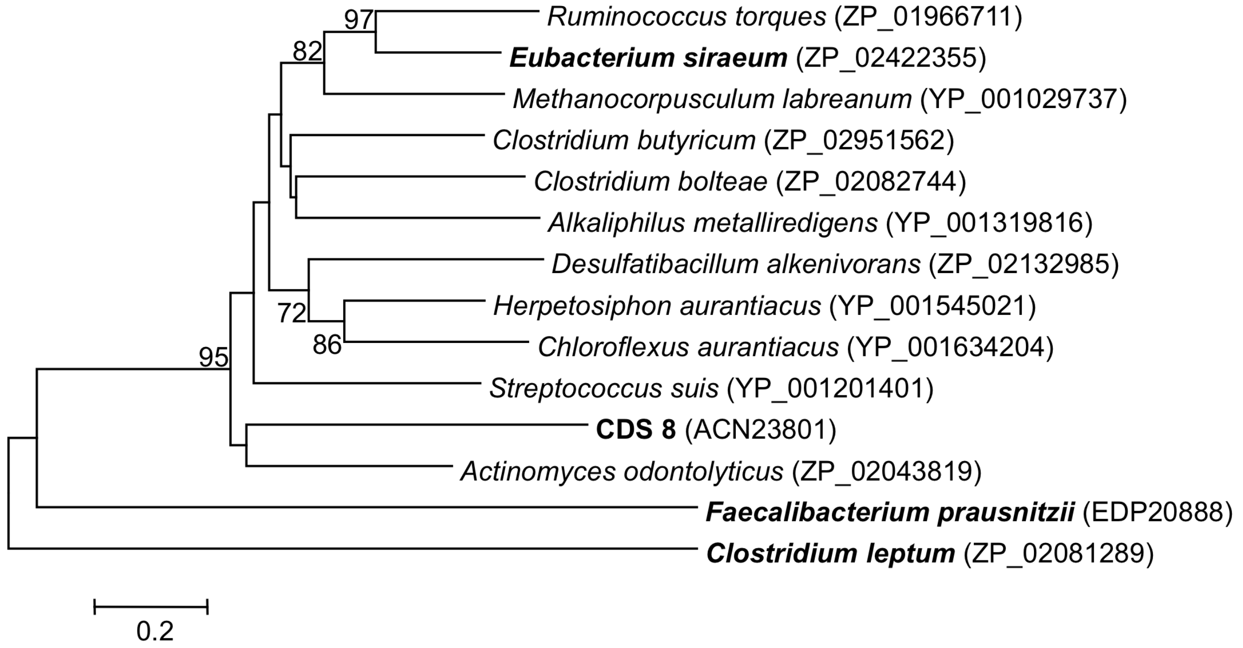

97
82
72
86
95
Ruminococcus torques (ZP_01966711)
Eubacterium siraeum (ZP_02422355)
Methanocorpusculum labreanum (YP_001029737)
Clostridium butyricum (ZP_02951562)
Clostridium bolteae (ZP_02082744)
Alkaliphilus metalliredigens (YP_001319816)
Desulfatibacillum alkenivorans (ZP_02132985)
Herpetosiphon aurantiacus (YP_001545021)
Chloroflexus aurantiacus (YP_001634204)
Streptococcus suis (YP_001201401)
CDS 8 (ACN23801)
Actinomyces odontolyticus (ZP_02043819)
Faecalibacterium prausnitzii (EDP20888)
Clostridium leptum (ZP_02081289)
0.2

e) CDS 9

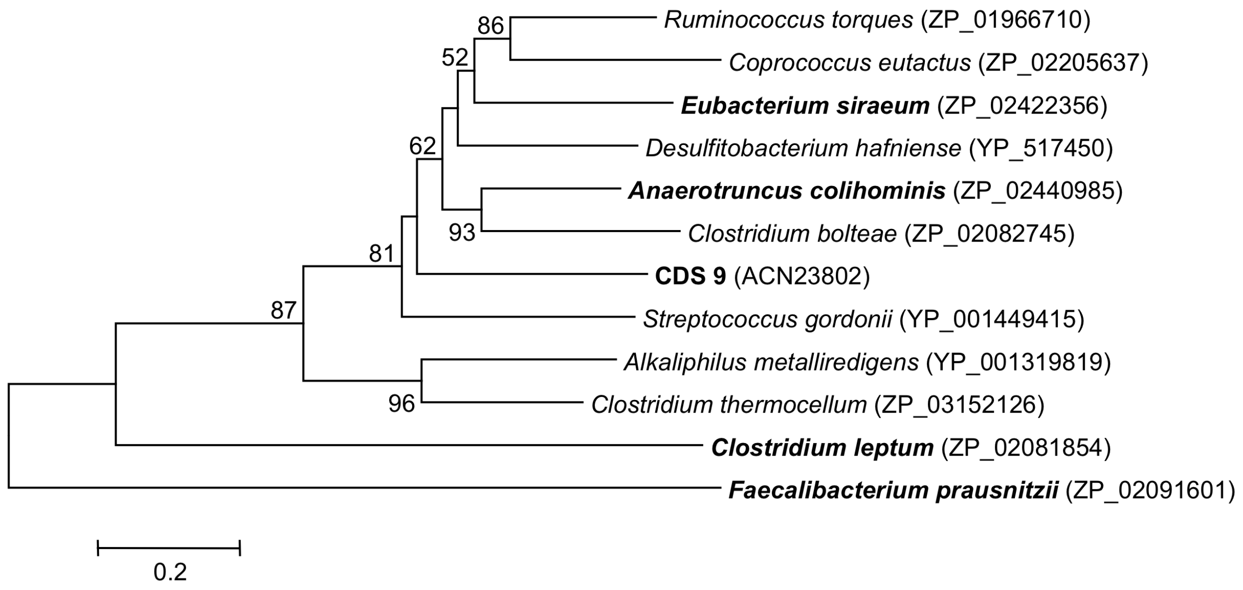

f) CDS 10

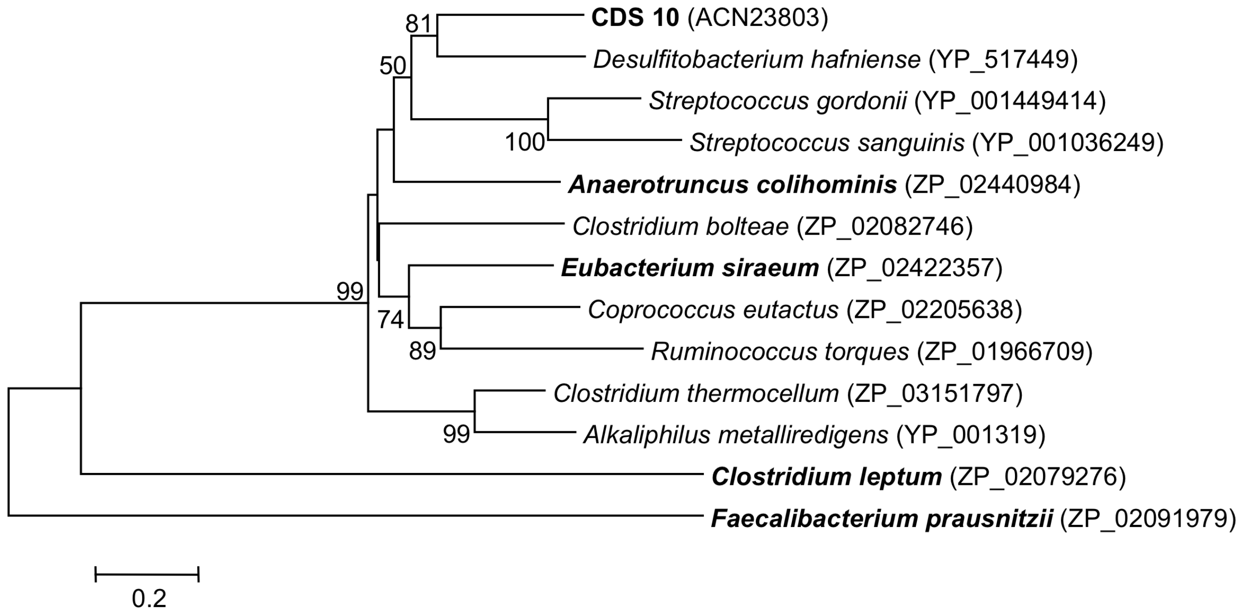

g) CDS 11

Neighbor joining

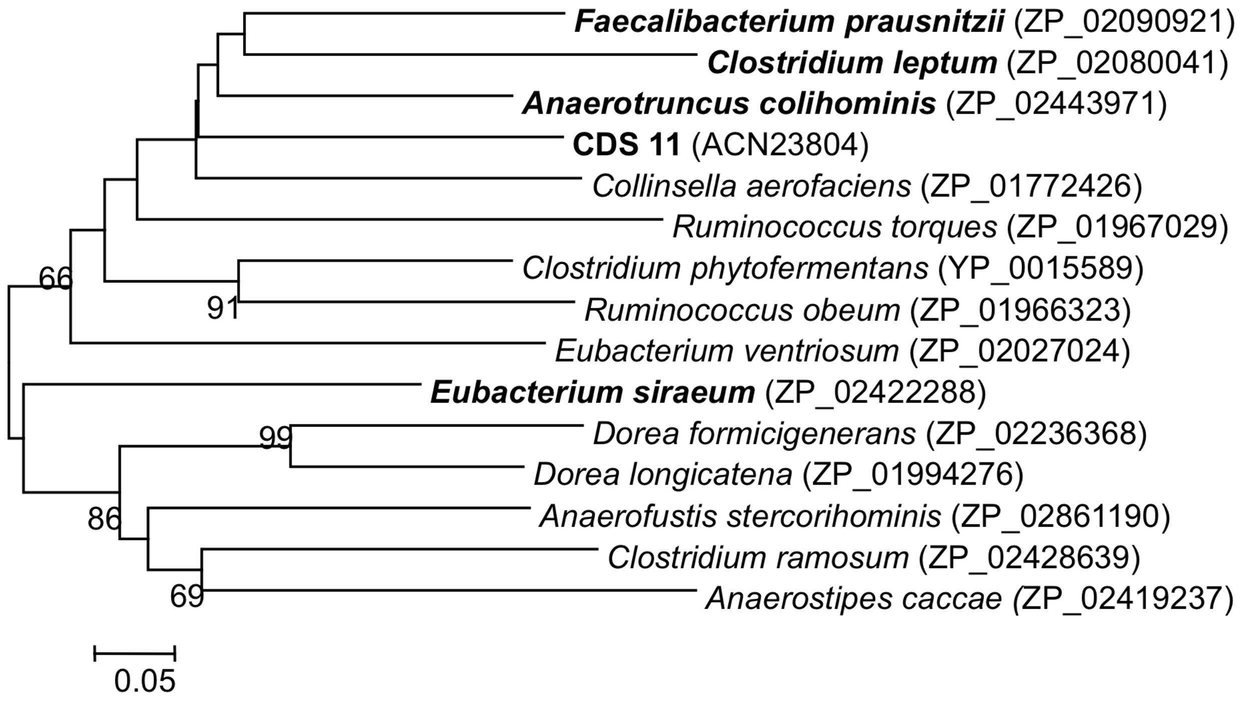

Maximum likelihood

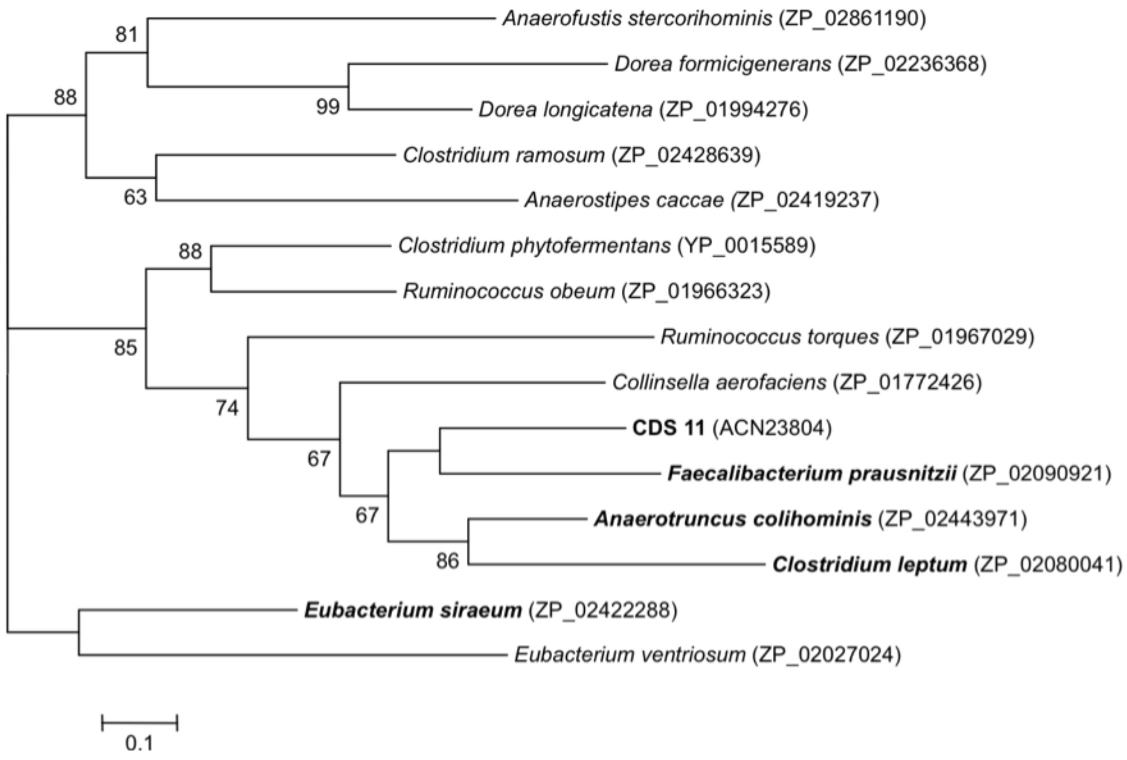

h) CDS 12

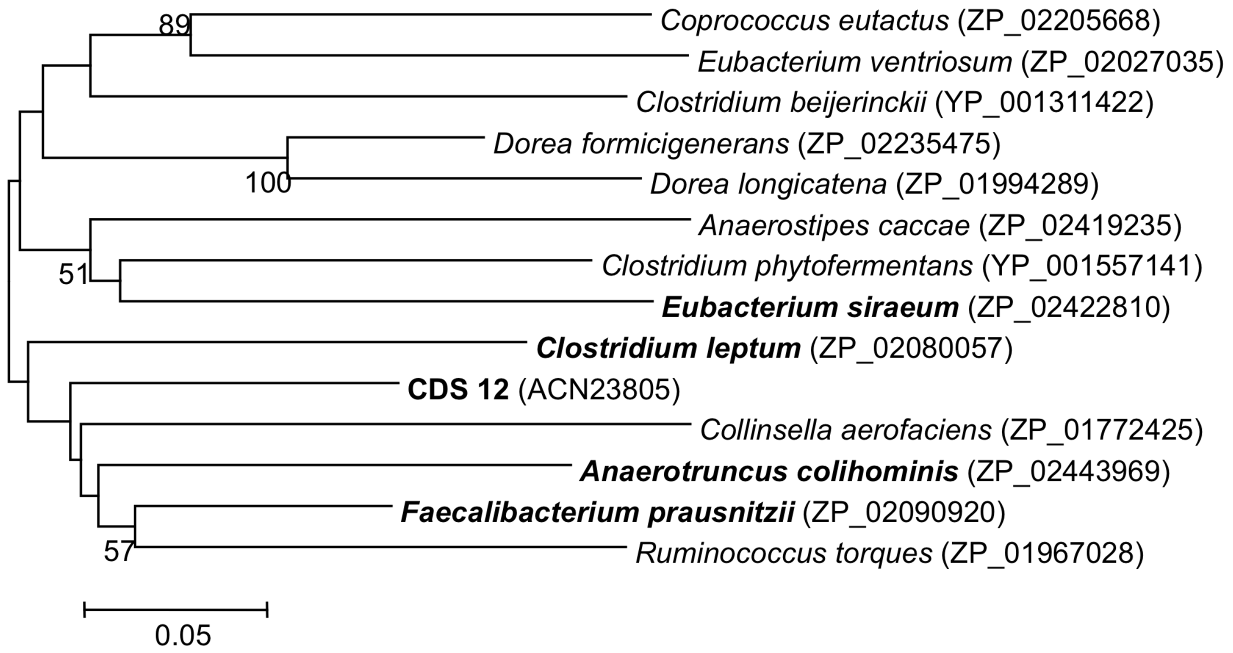

i) CDS 13

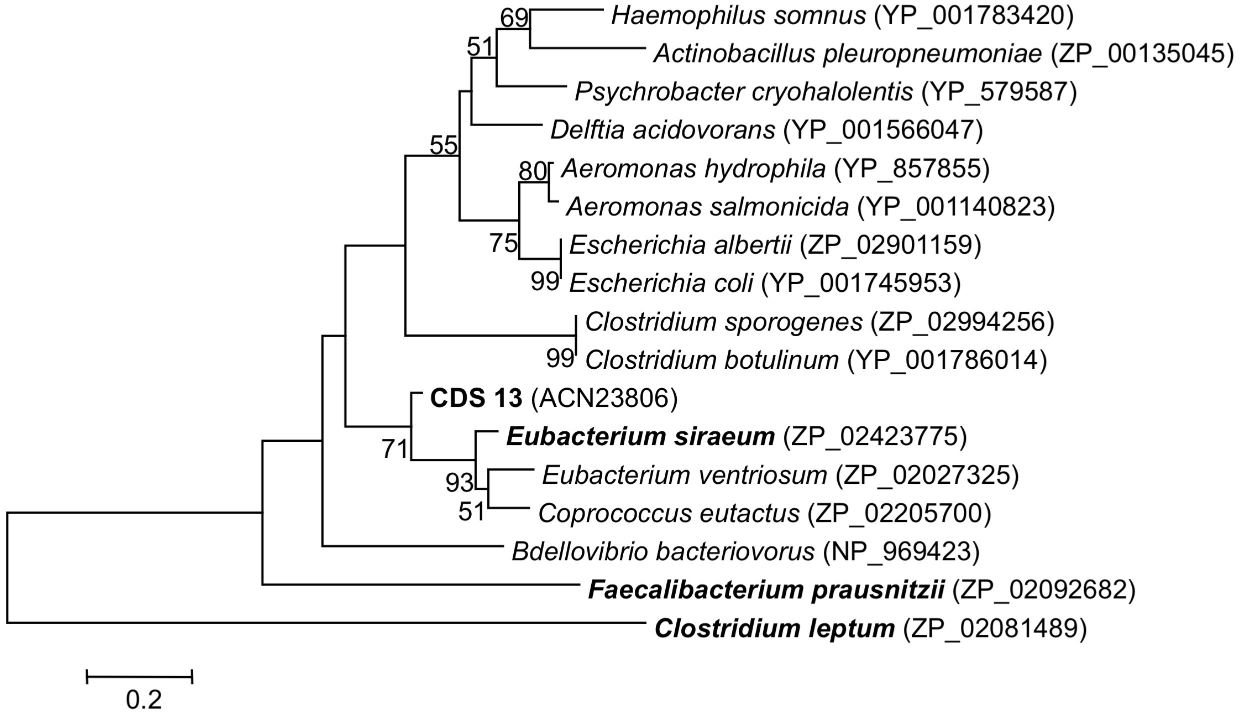

j) CDS 14

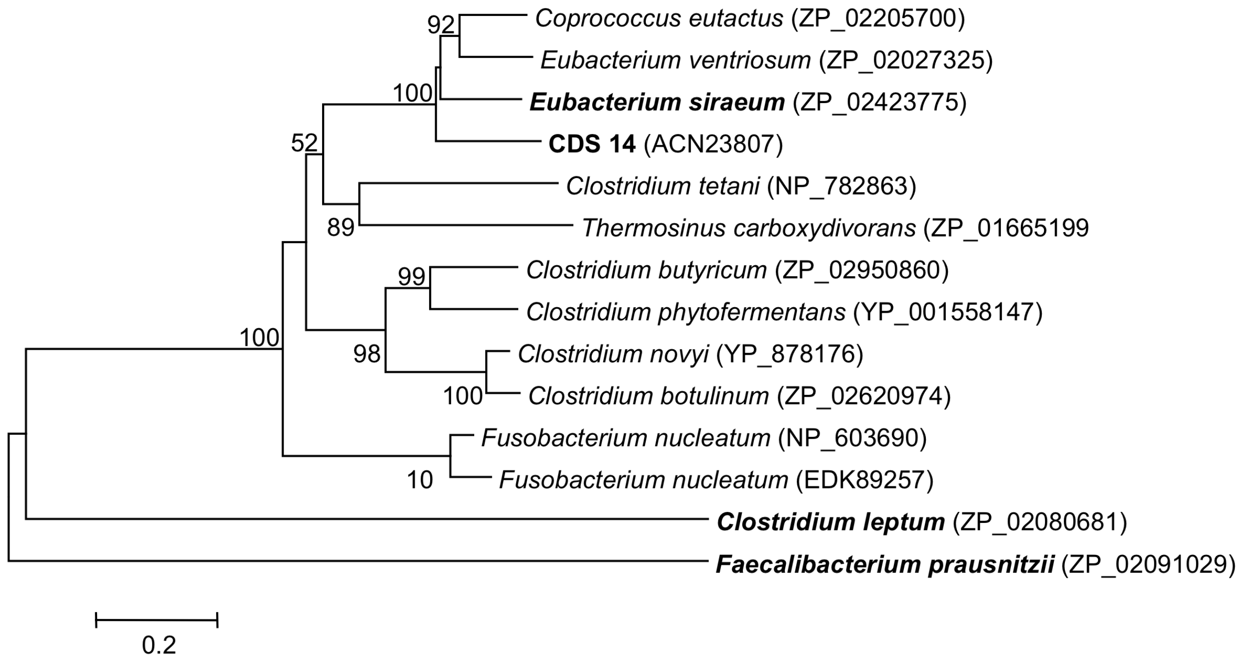

k) CDS 15

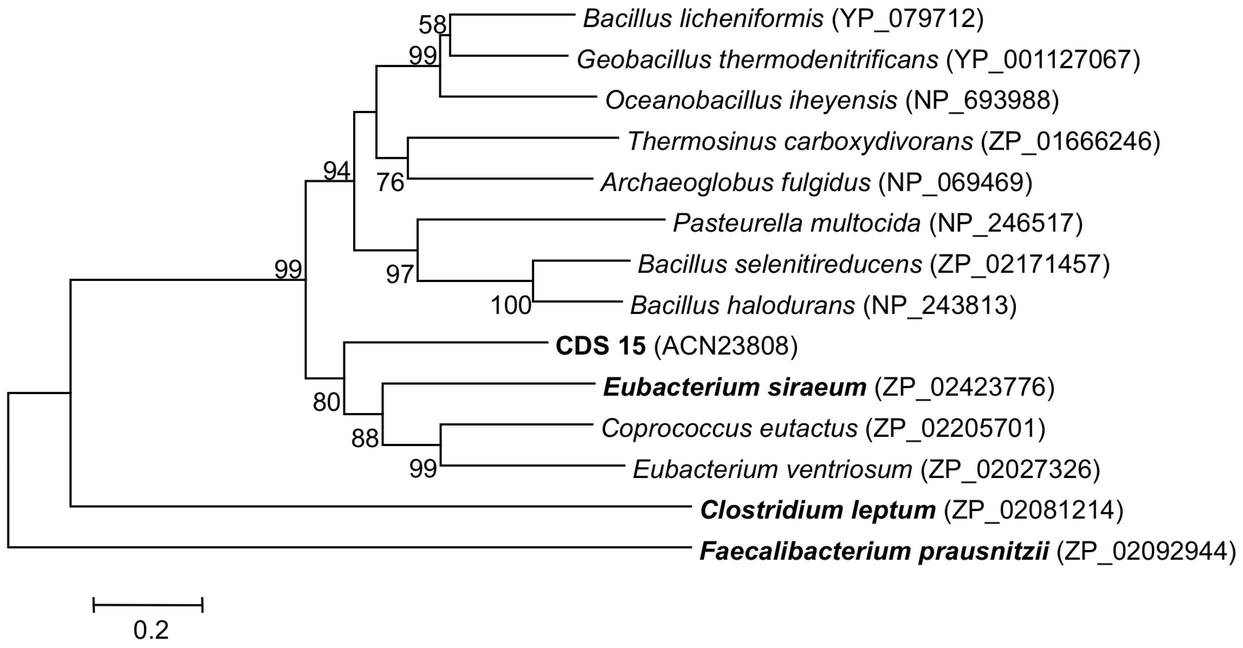

l) CDS 16

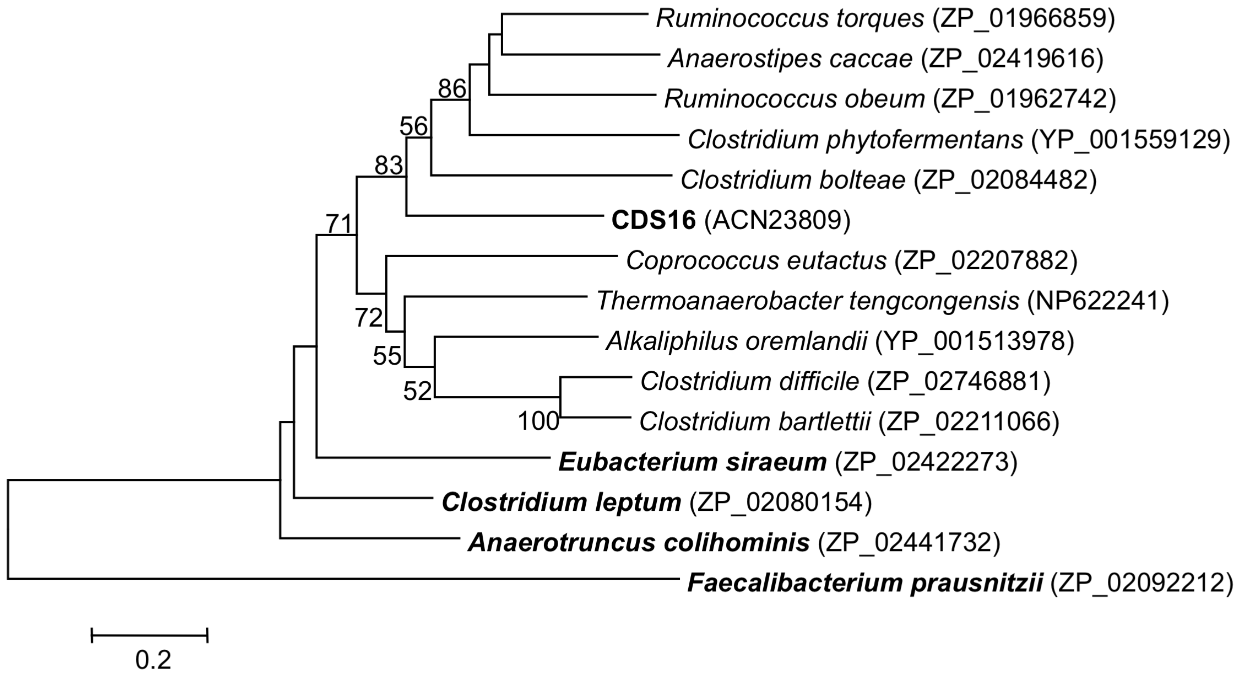

m) CDS 17

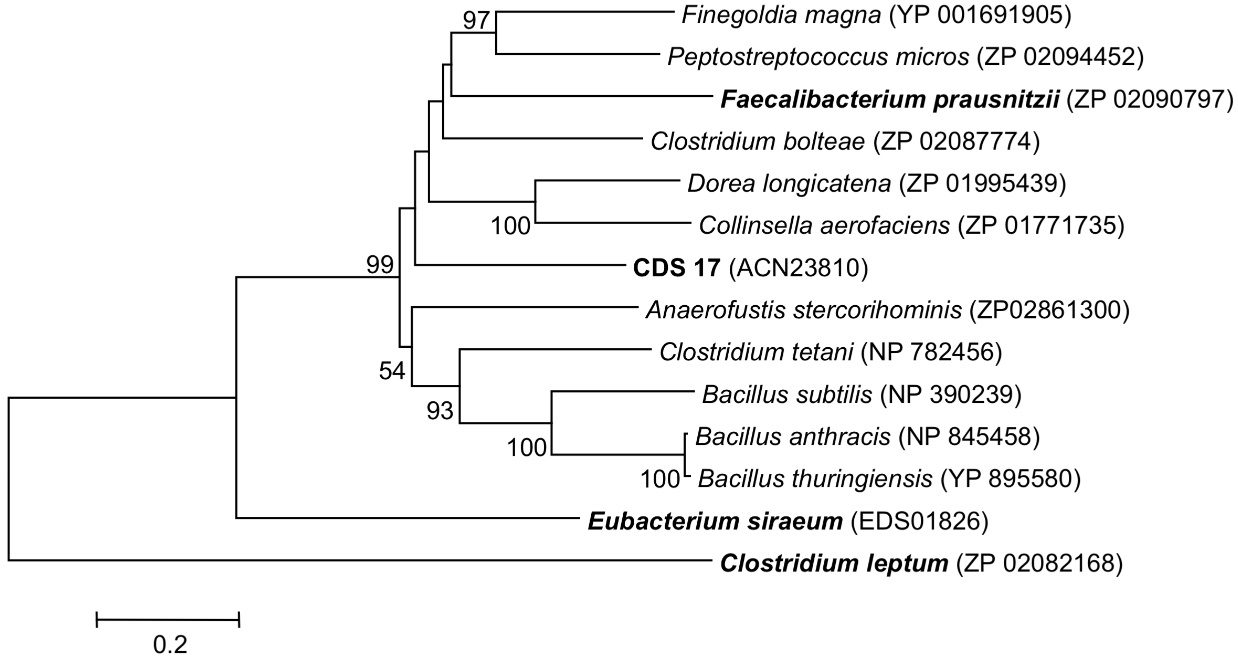

n) CDS 18

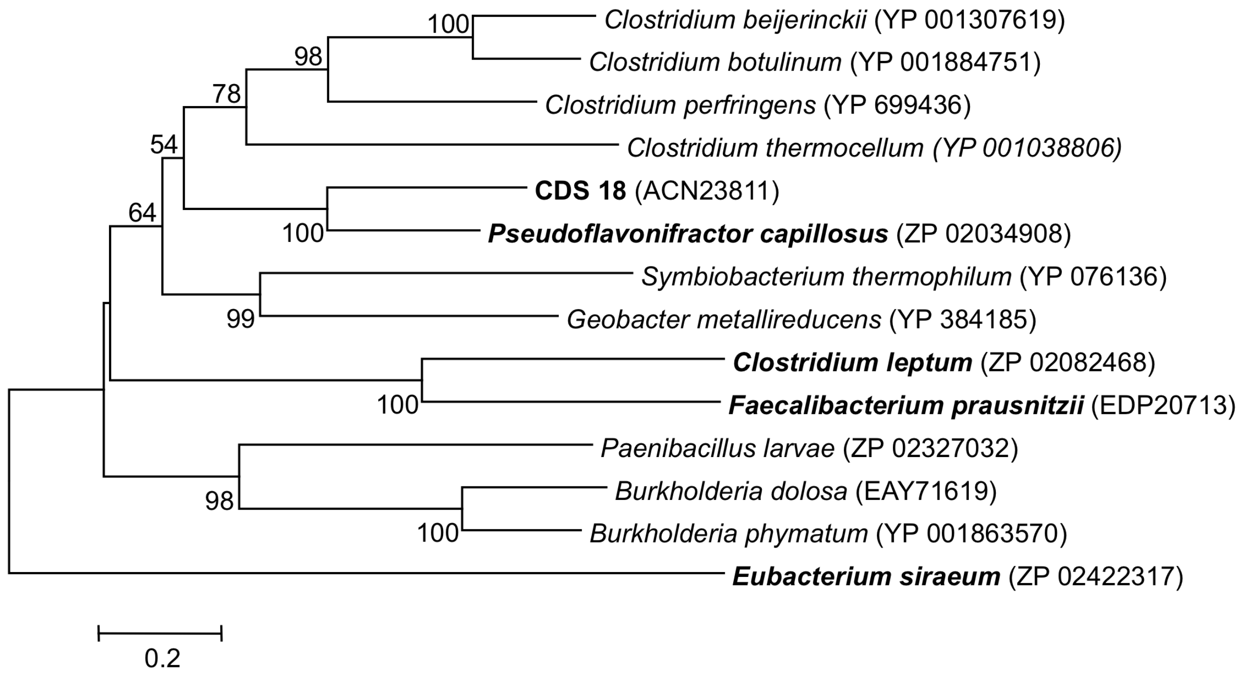

o) CDS 19

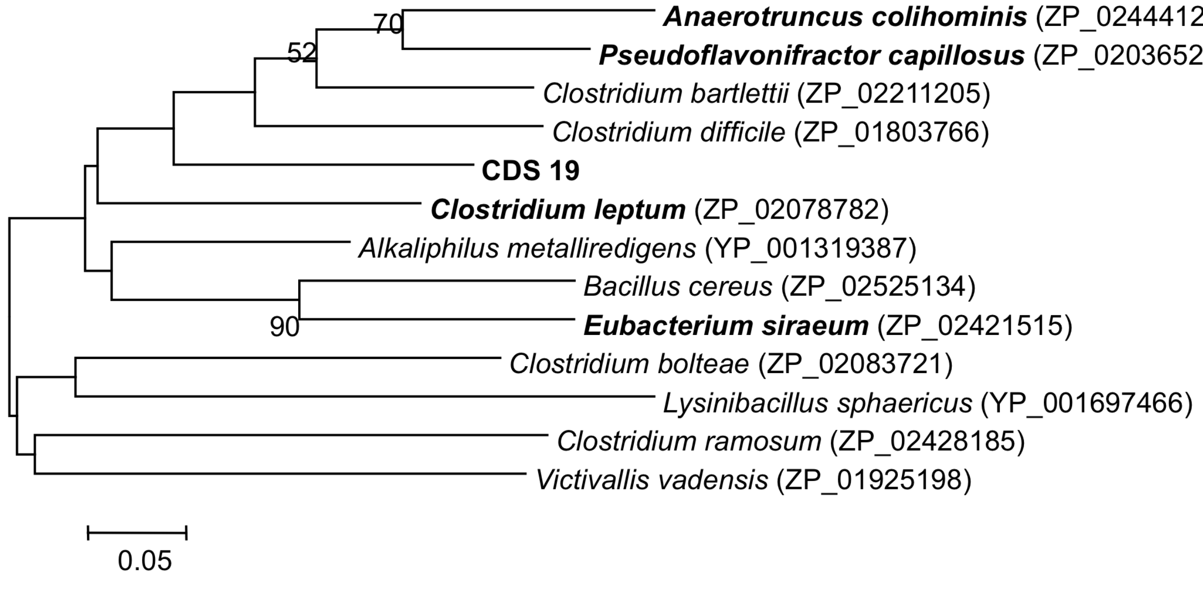

p) CDS 20

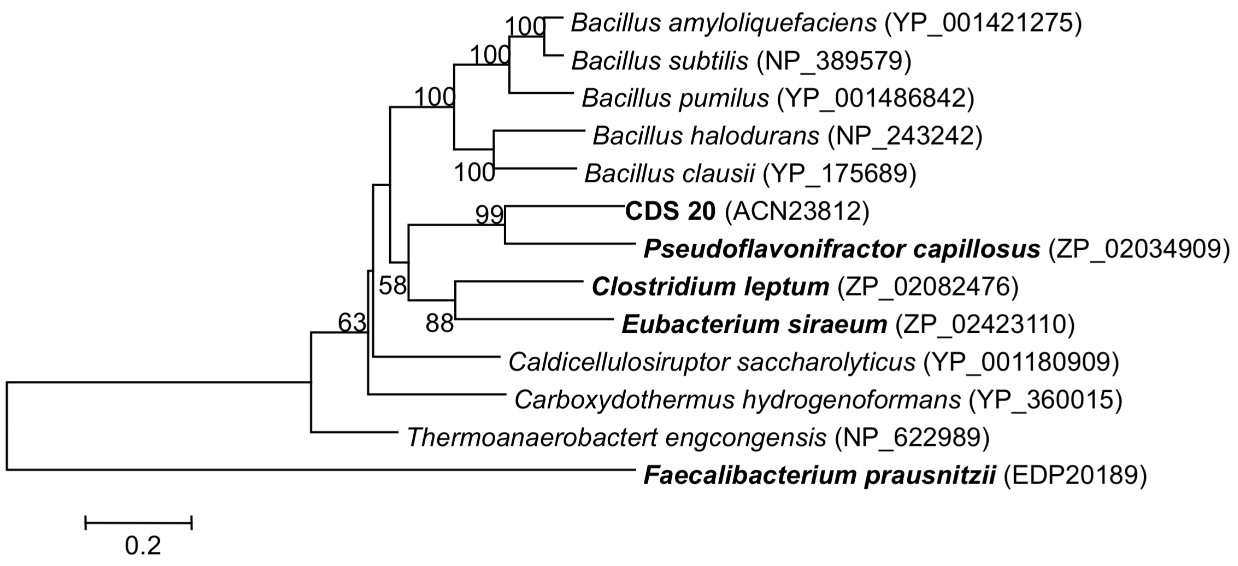

q) CDS 21

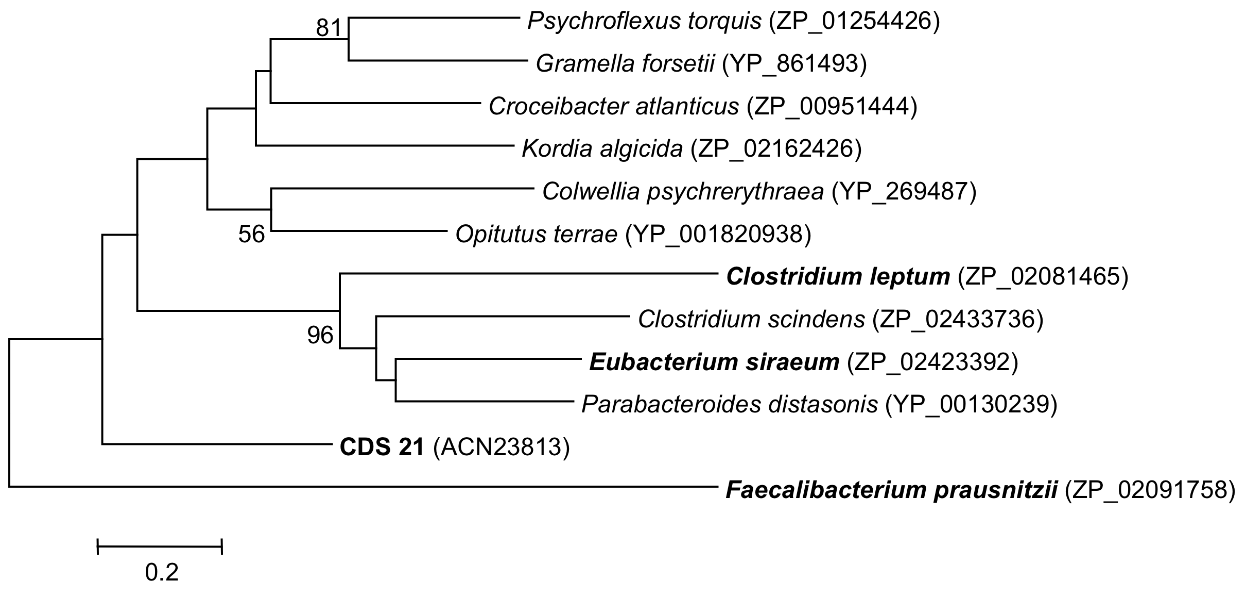

r) CDS 22

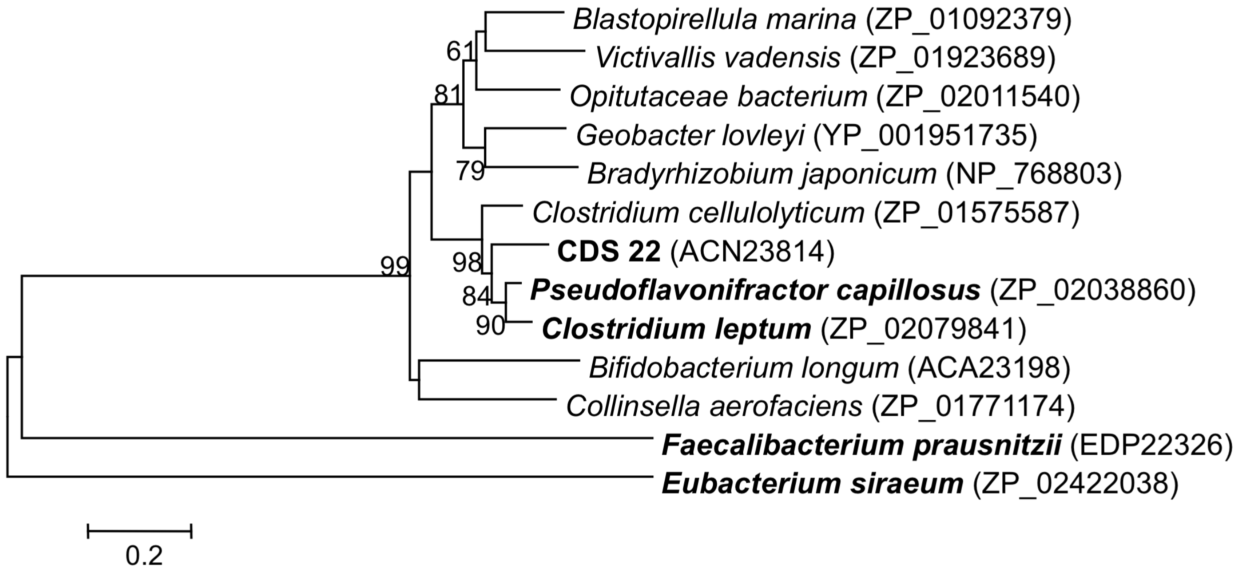

s) CDS 29

Neighbor joining

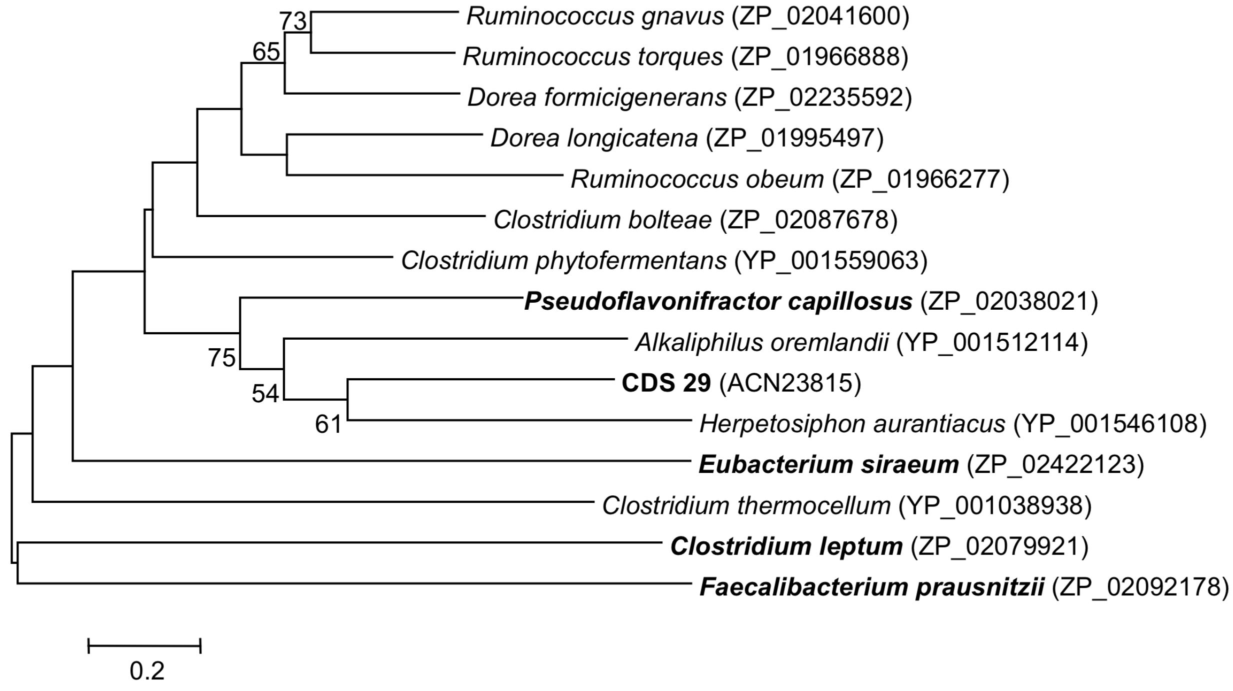

Maximum likelihood

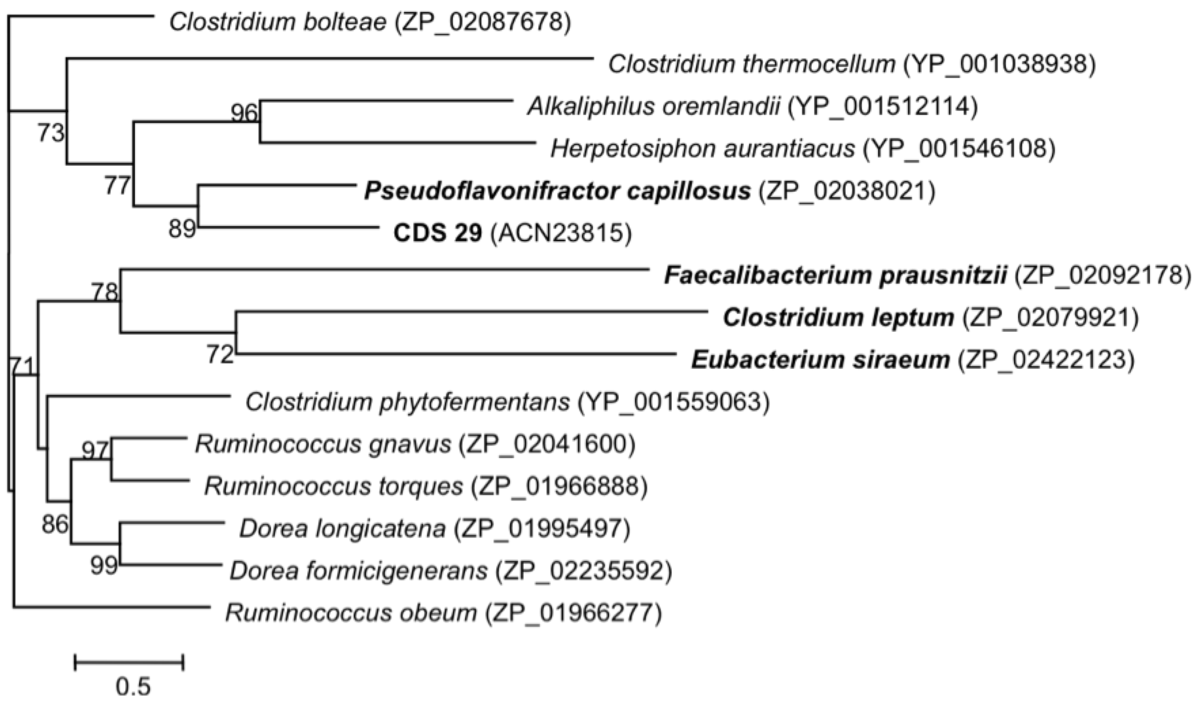

t) CDS 30

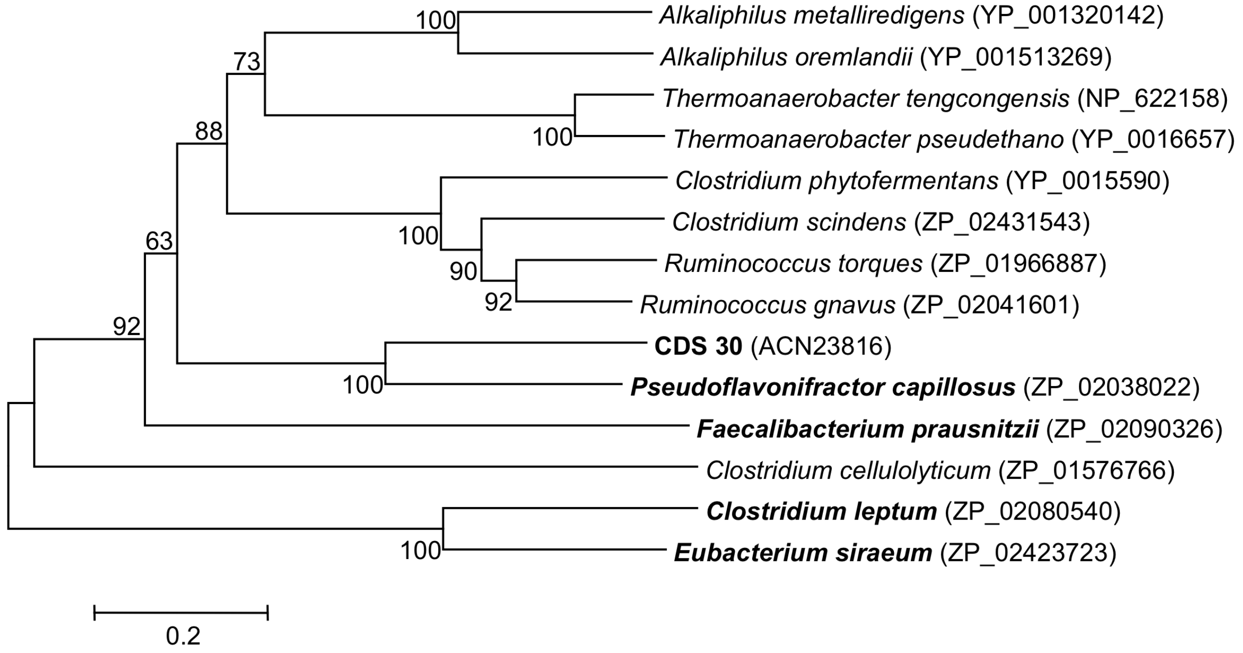

u) CDS 31

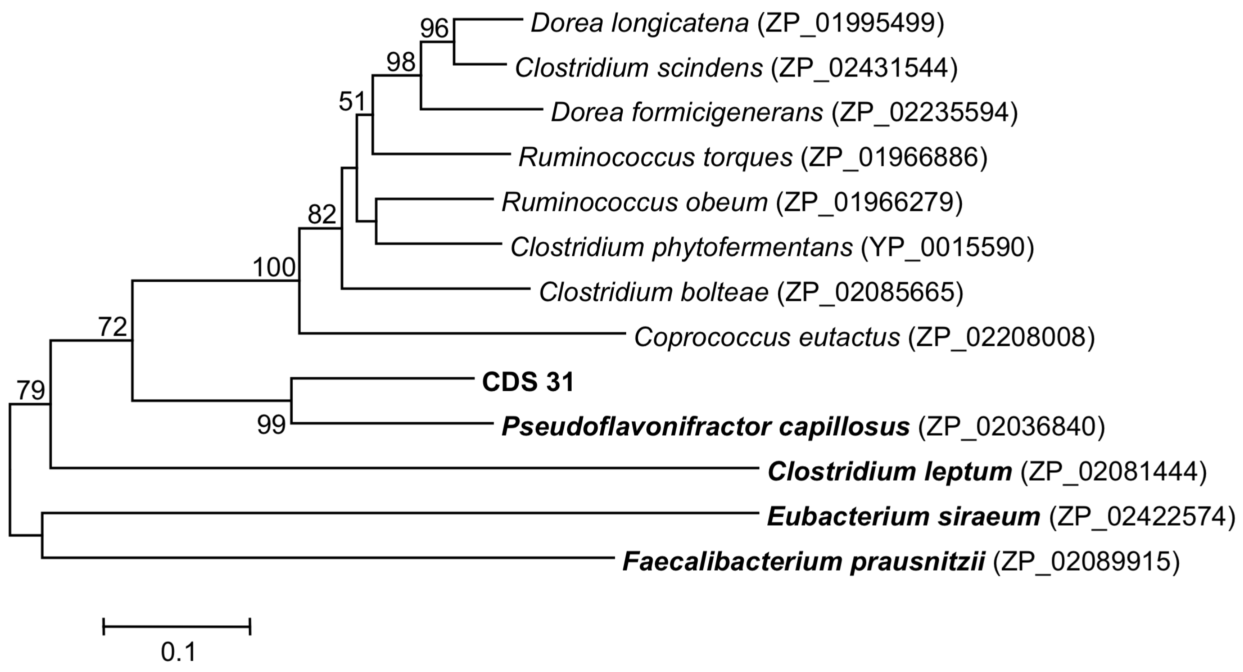

v) CDS 32

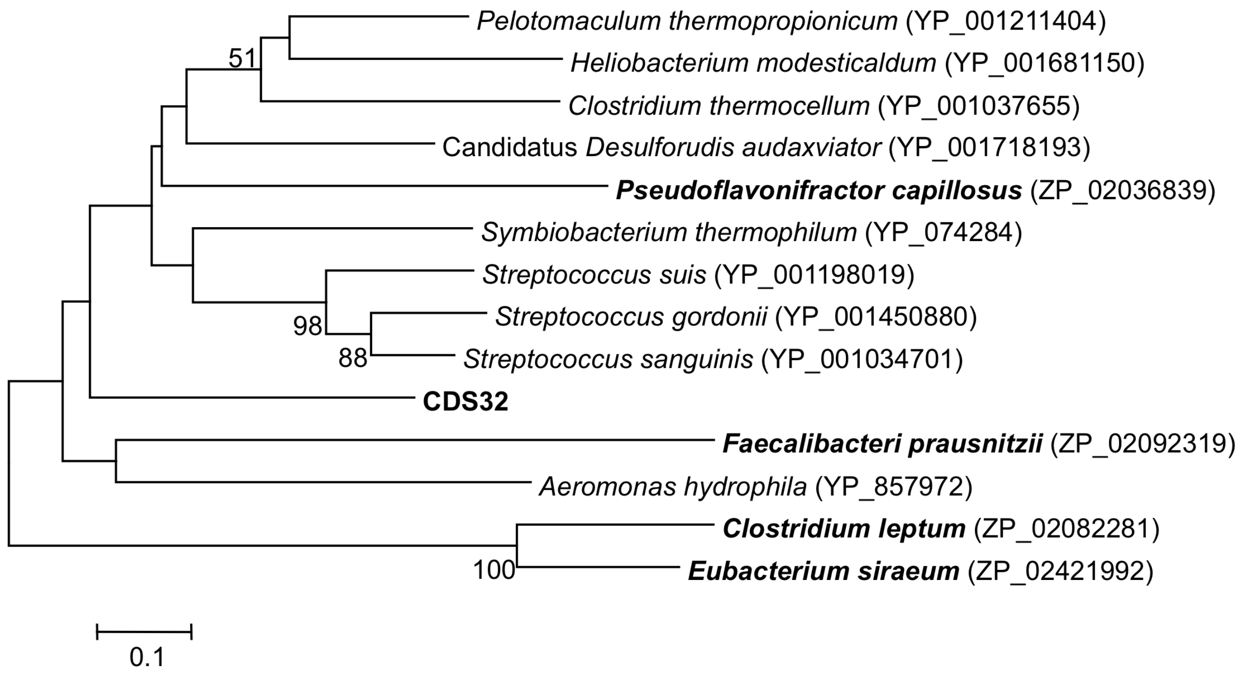

w) CDS 33

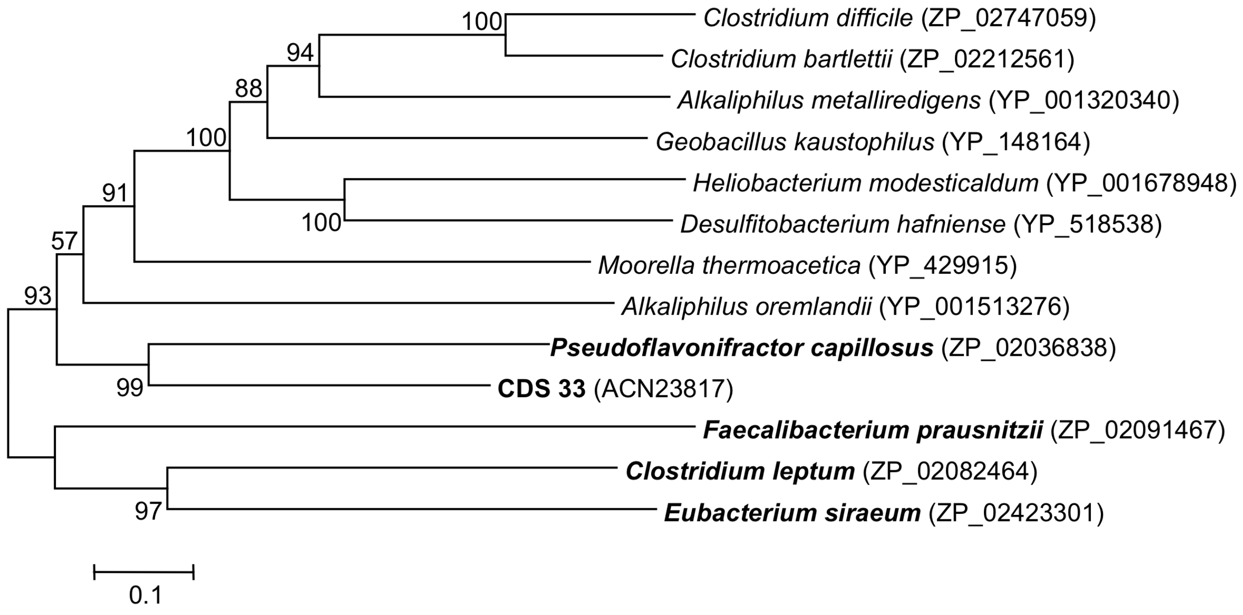

x) CDS 34

Neighbor joining

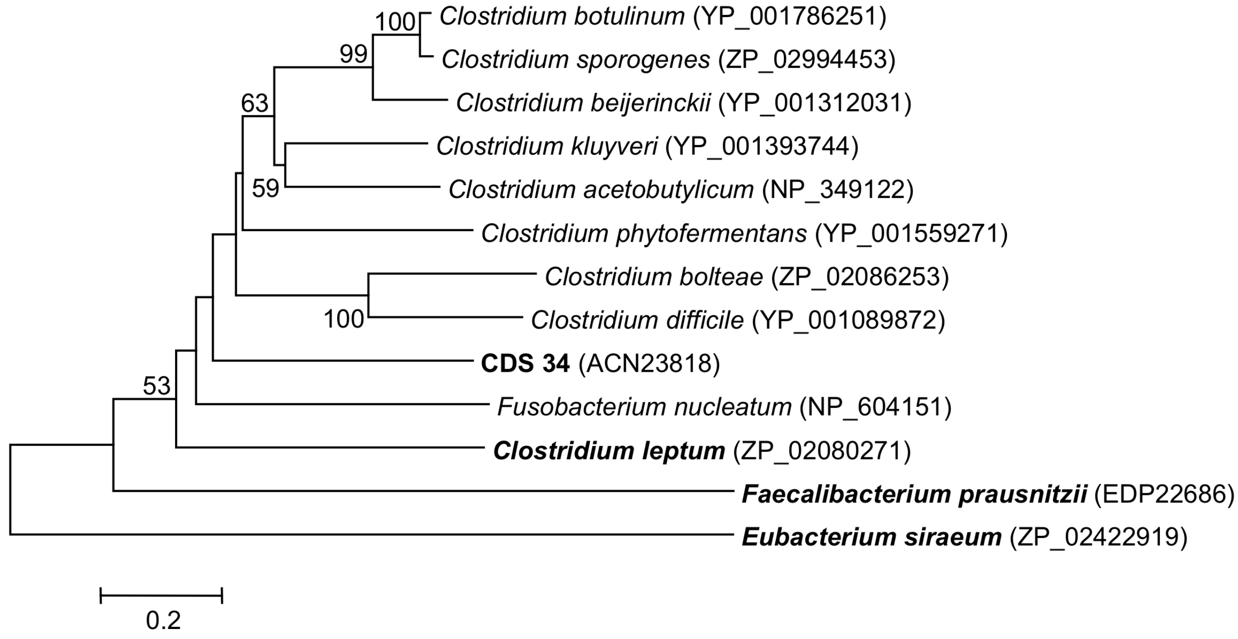

Maximum likelihood

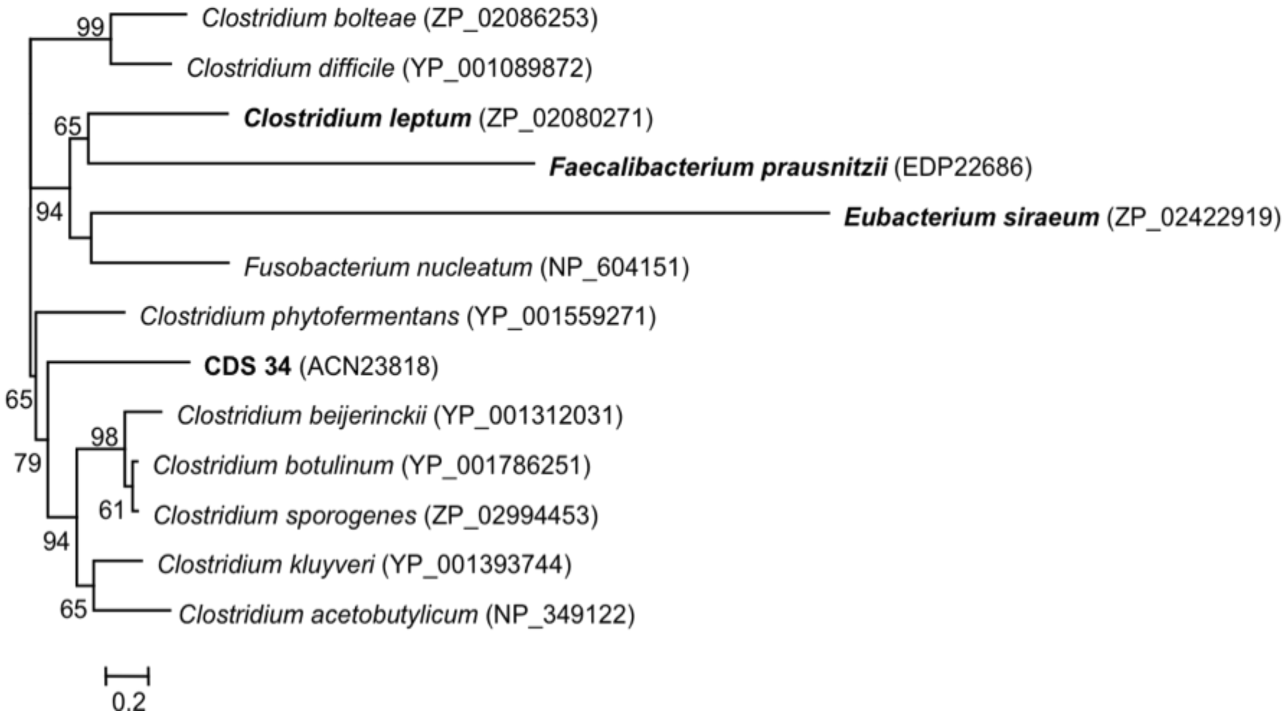

Supplement: Figure S2 — Neighbor-joining phylogenetic trees of amino acid sequences from fosmid 7–14 that were used to assess LGT, as described in the text. Sequences in bold represent those from Clostridium cluster IV. For CDS 11, 29, and 34 the conclusion of LGT based upon the neighbor-joining trees differed from that based upon maximum likelihood trees (as listed in Table 1). Thus for these CDSs we also show the maximum likelihood trees. (4.49 MB PDF) [file pone.0010785.s002.pdf]
