# Supplementary material for: Phylogenetic Evidence for Lateral Gene Transfer in the Intestine of Marine Iguanas
Source: PLoS One. 2010 May 24;5(5):e10785. doi: 10.1371/journal.pone.0010785 (PMC2875401; doi:10.1371/journal.pone.0010785)

Figure S3

a) CDS 1

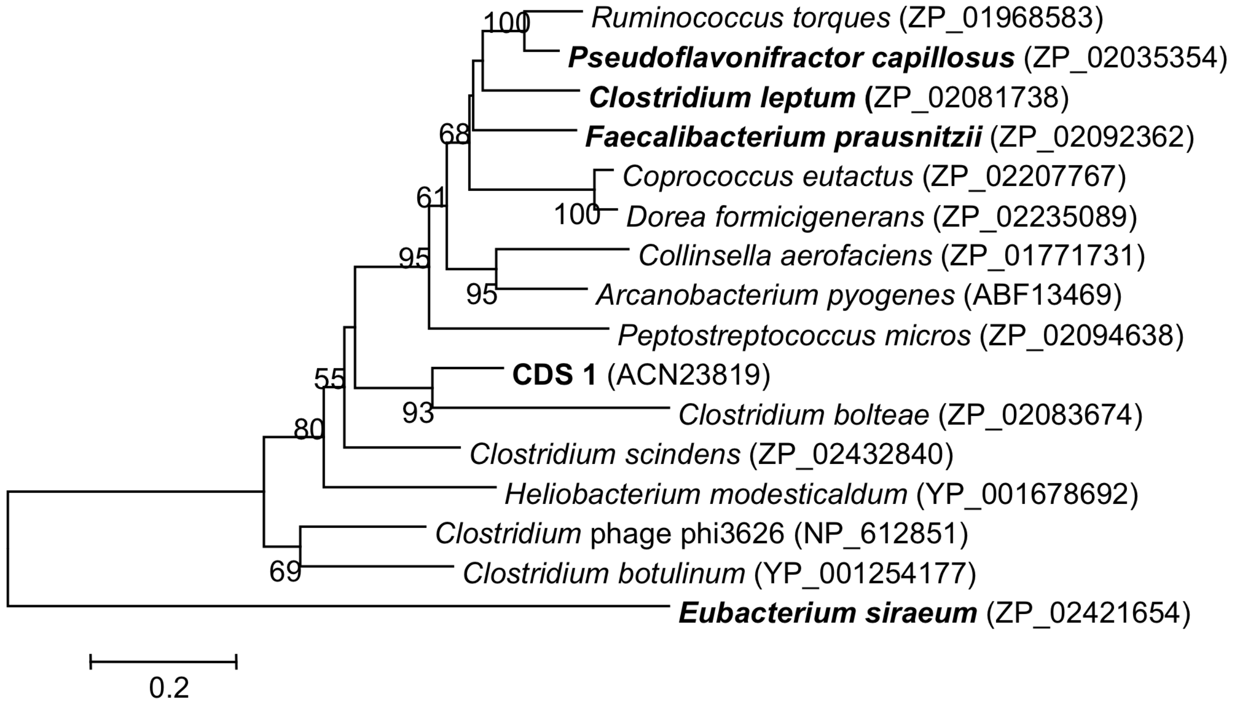

b) CDS 2

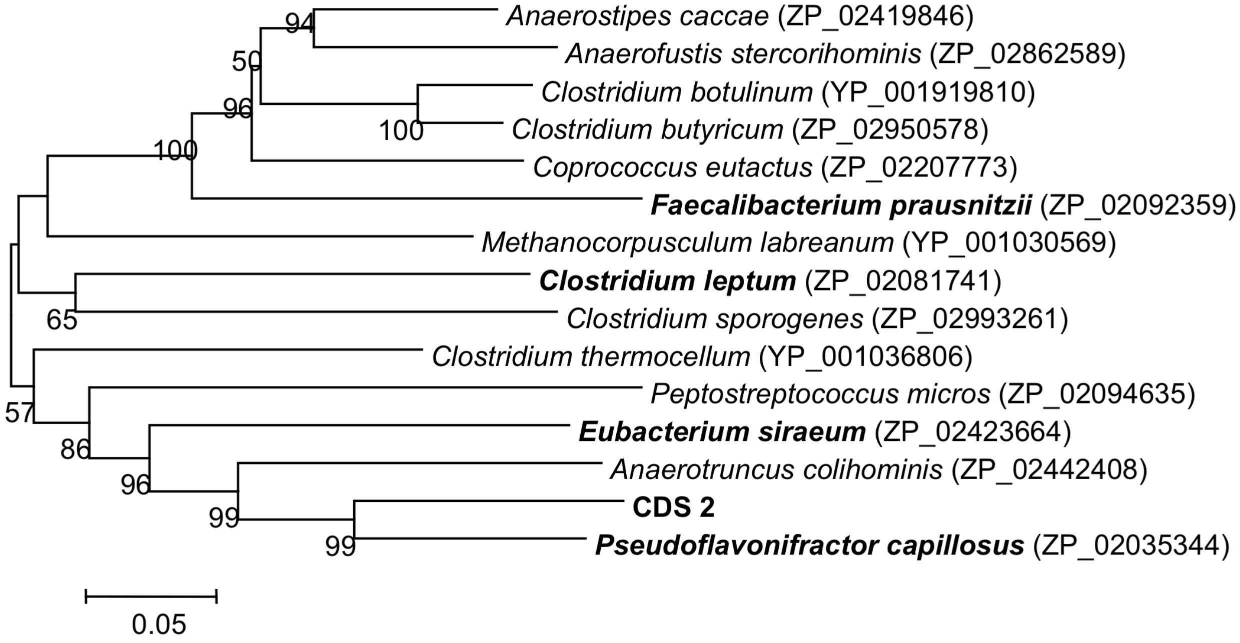

c) CDS 3

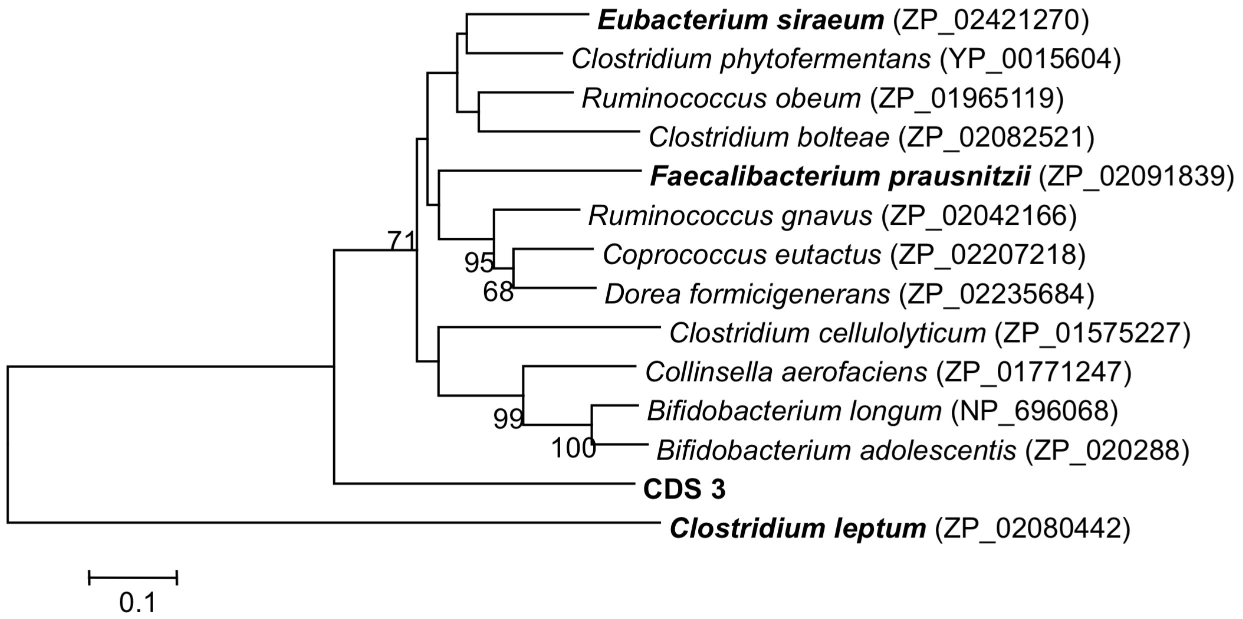

Eubacterium siraeum (ZP_02421270)
Clostridium phytofermentans (YP_0015604)
Ruminococcus obeum (ZP_01965119)
Clostridium bolteae (ZP_02082521)
Faecalibacterium prausnitzii (ZP_02091839)
Ruminococcus gnavus (ZP_02042166)
Coprococcus eutactus (ZP_02207218)
Dorea formicigenerans (ZP_02235684)
Clostridium cellulolyticum (ZP_01575227)
Collinsella aerofaciens (ZP_01771247)
Bifidobacterium longum (NP_696068)
Bifidobacterium adolescentis (ZP_020288)
CDS 3
Clostridium leptum (ZP_02080442)
71
95
68
99
100
0.1

d) CDS 4

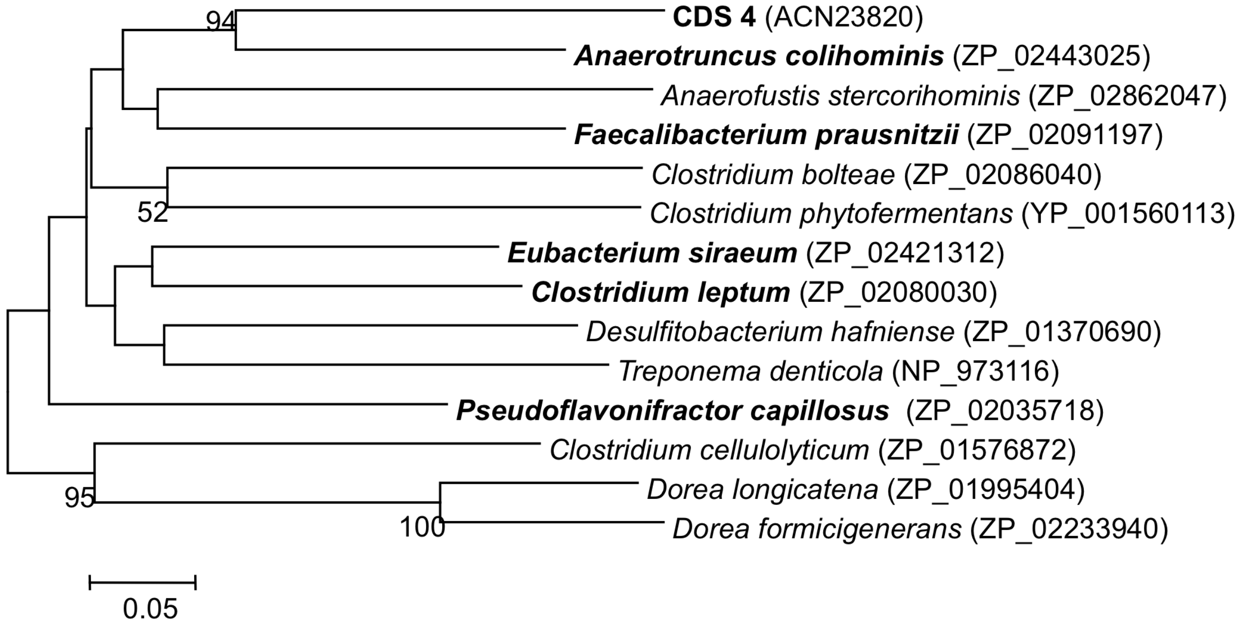

CDS 4 (ACN23820)
Anaerotruncus colihominis (ZP_02443025)
Anaerofustis stercorihominis (ZP_02862047)
Faecalibacterium prausnitzii (ZP_02091197)
Clostridium bolteae (ZP_02086040)
Clostridium phytofermentans (YP_001560113)
Eubacterium siraeum (ZP_02421312)
Clostridium leptum (ZP_02080030)
Desulfitobacterium hafniense (ZP_01370690)
Treponema denticola (NP_973116)
Pseudoflavonifractor capillosus (ZP_02035718)
Clostridium cellulolyticum (ZP_01576872)
Dorea longicatena (ZP_01995404)
Dorea formicigenerans (ZP_02233940)
94
52
95
100
0.05

e) CDS 5

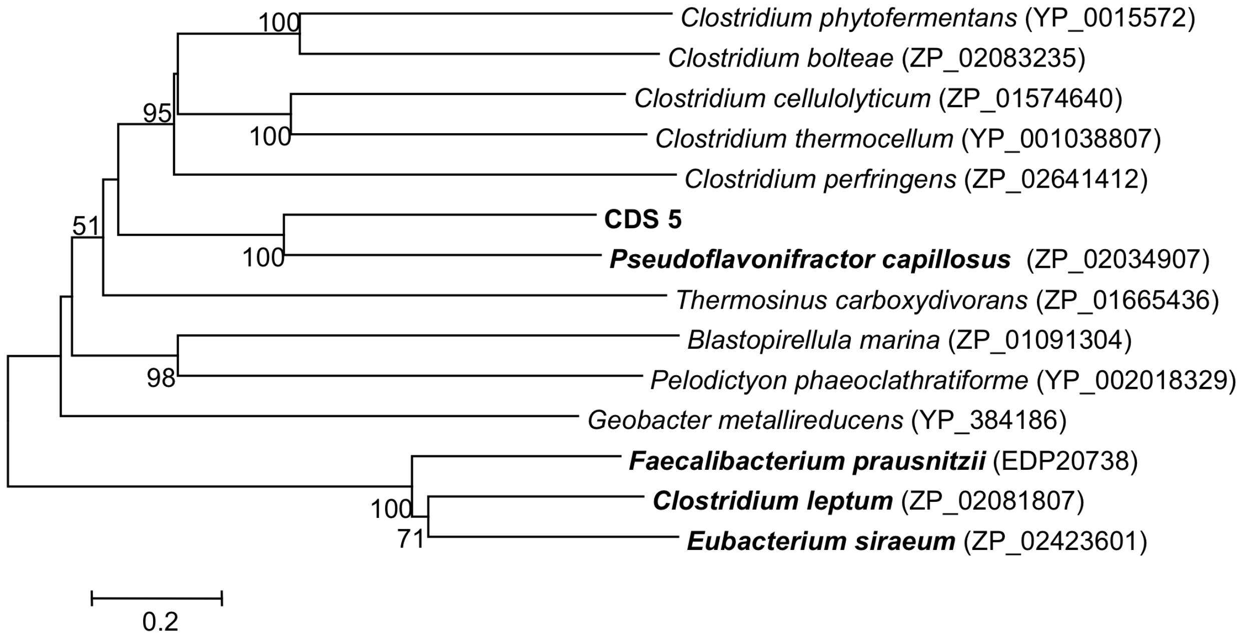

f) CDS 17

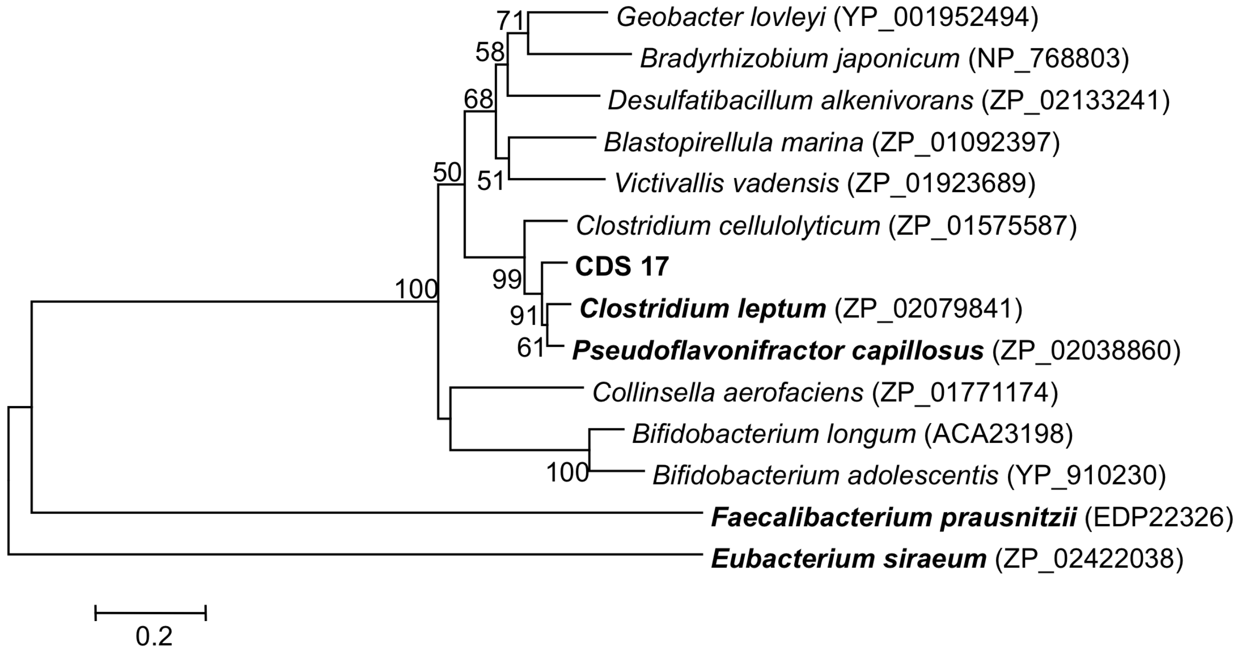

g) CDS 18

Neighbor joining

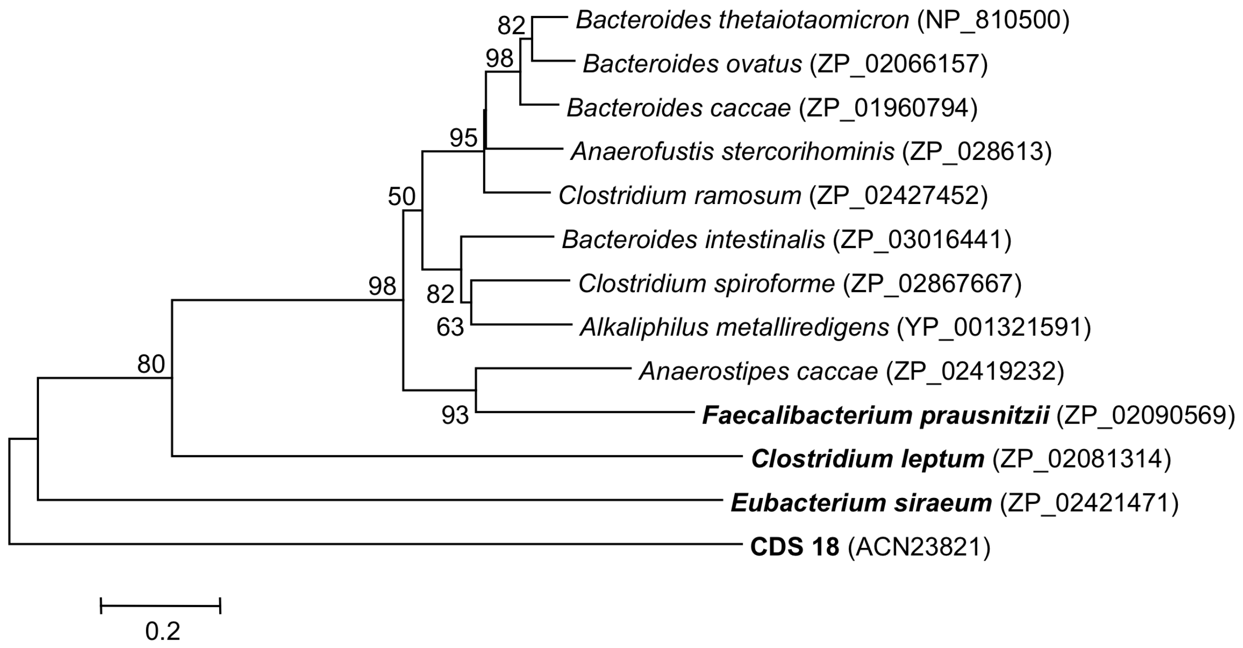

Maximum likelihood

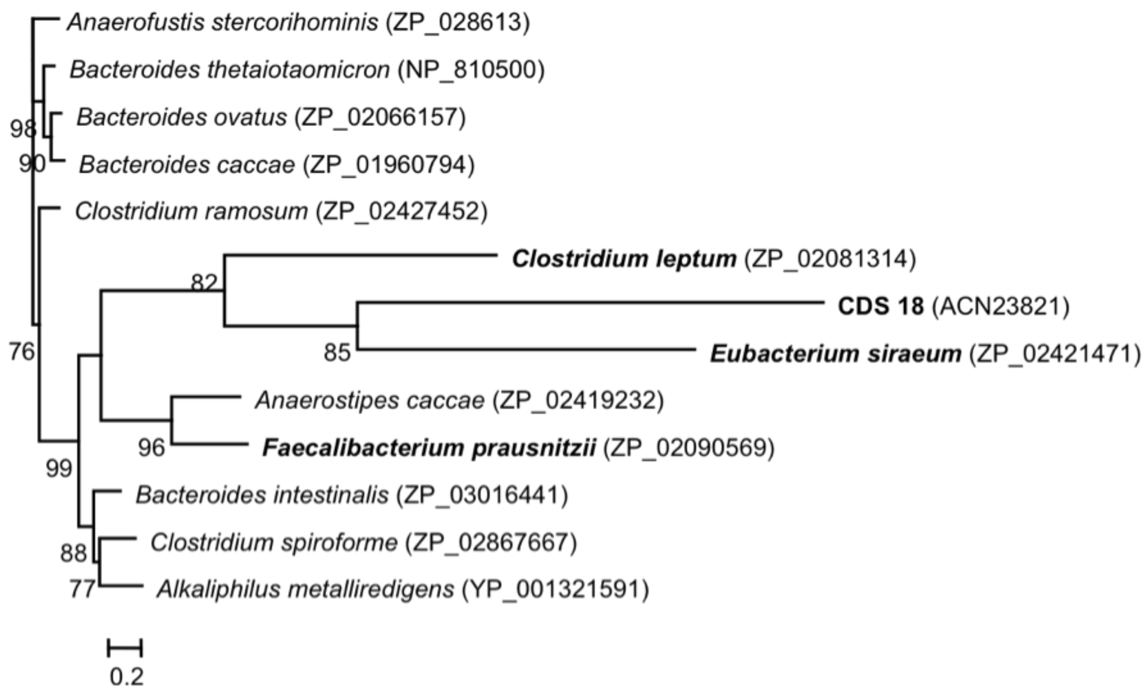

h) CDS 19

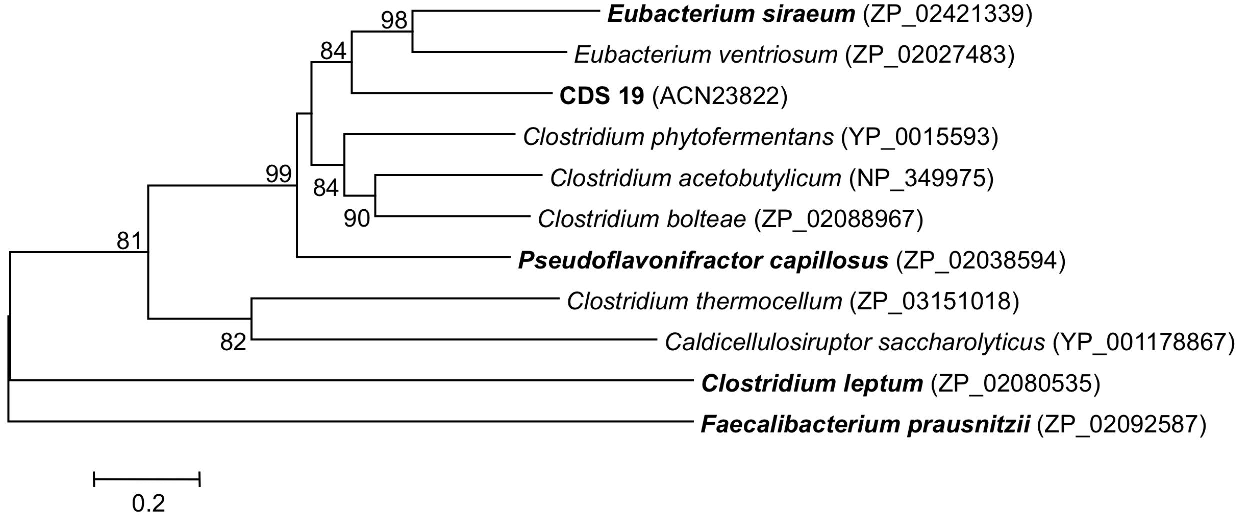

i) CDS 20

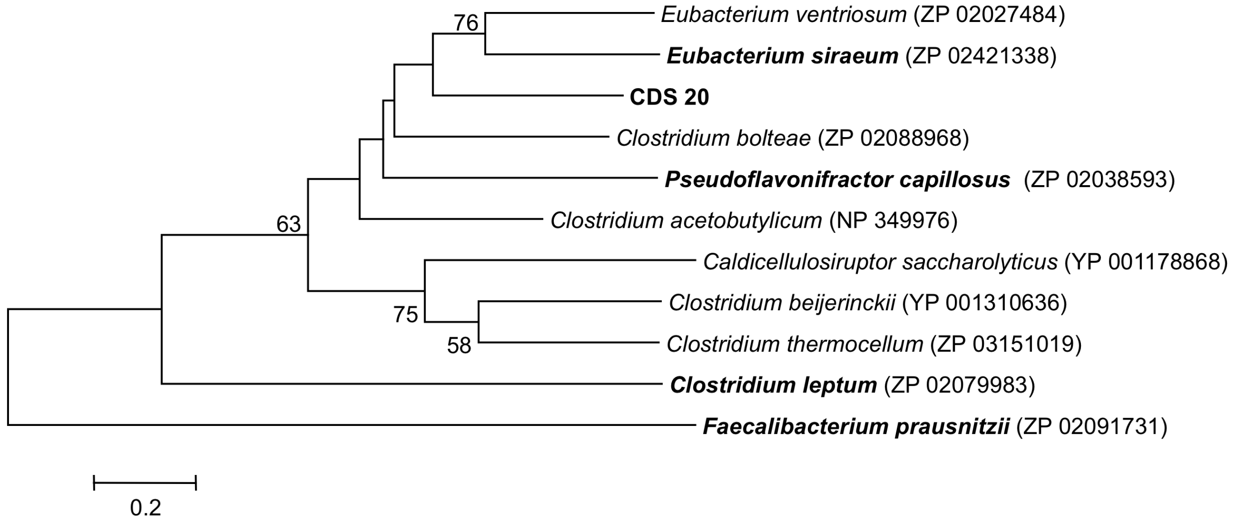

j) CDS 21

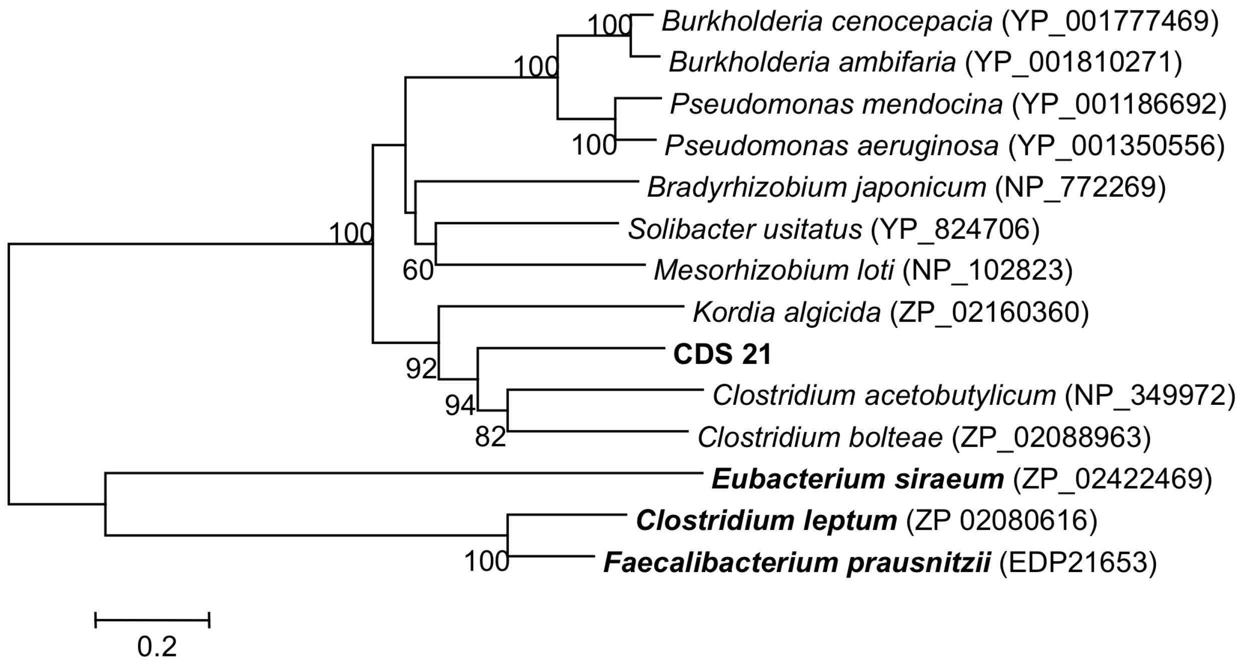

k) CDS 22

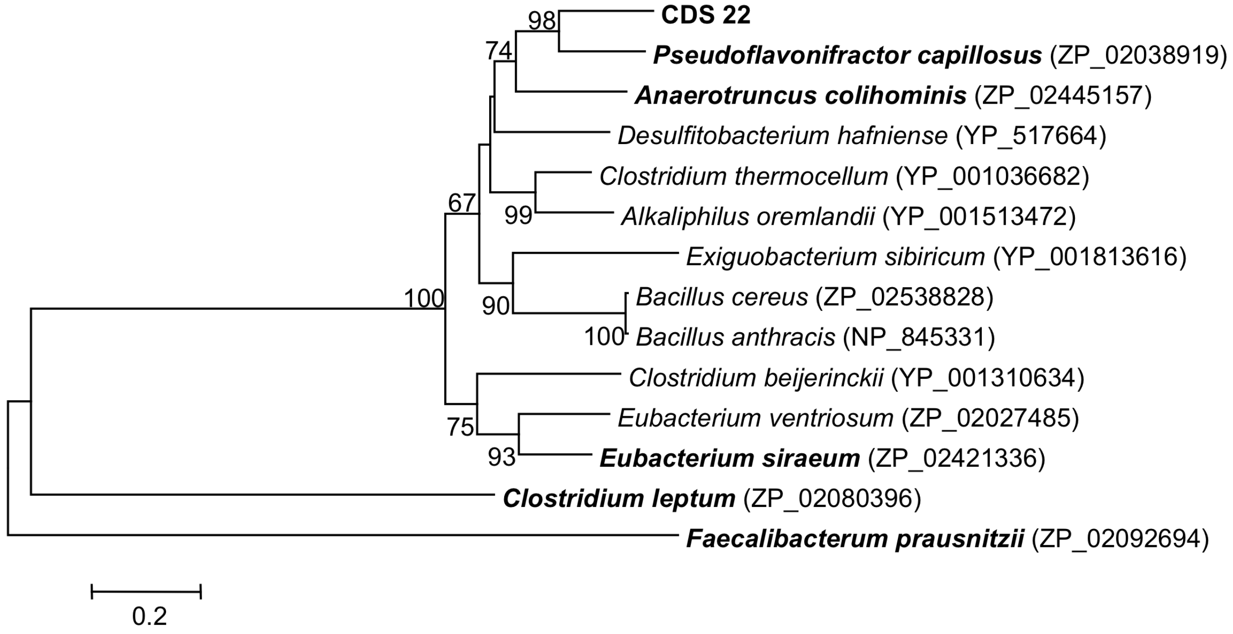

l) CDS 23

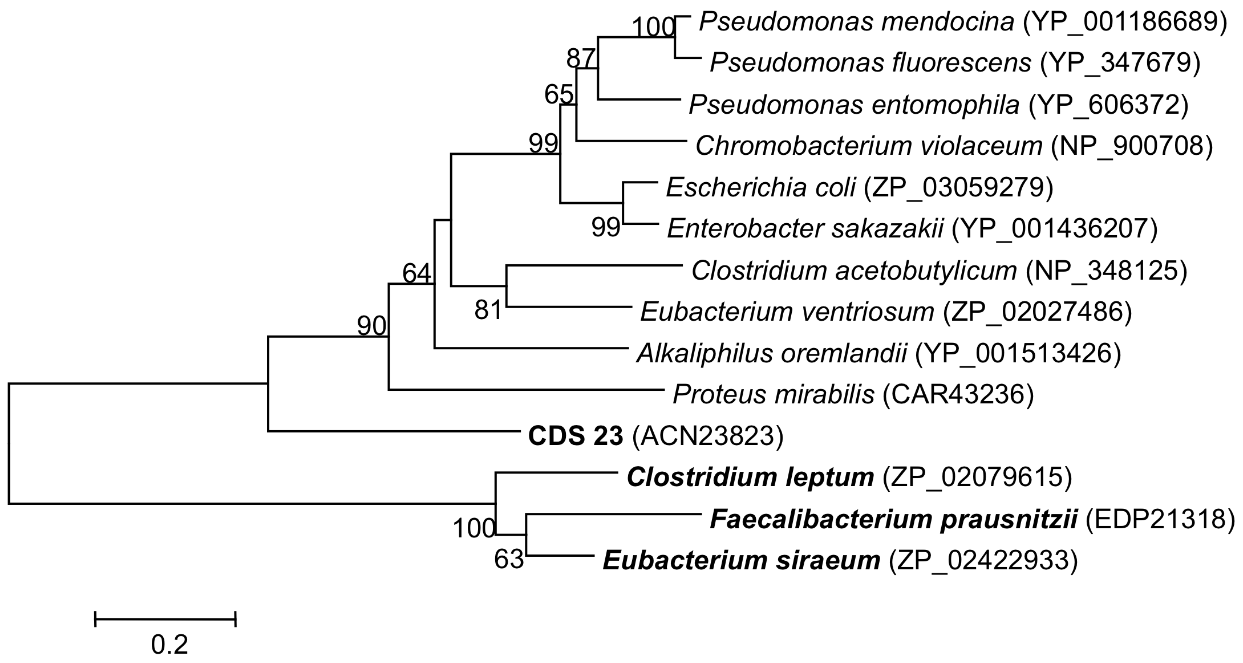

m) CDS 24

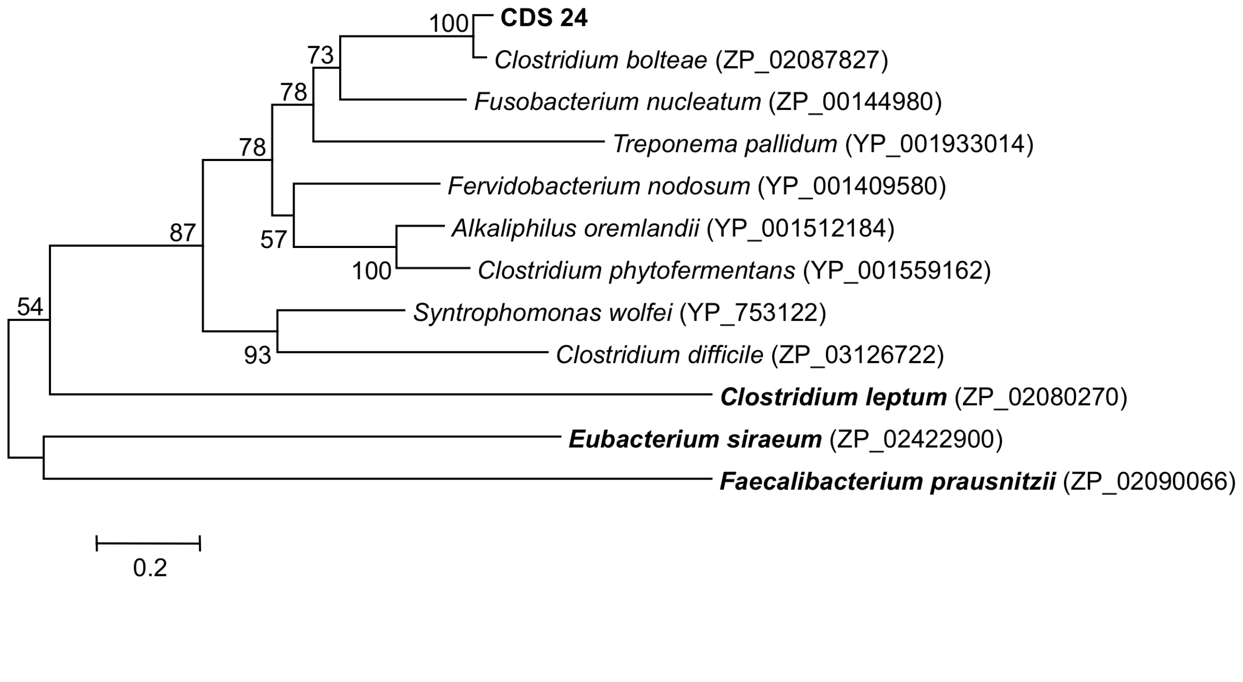

n) CDS 25

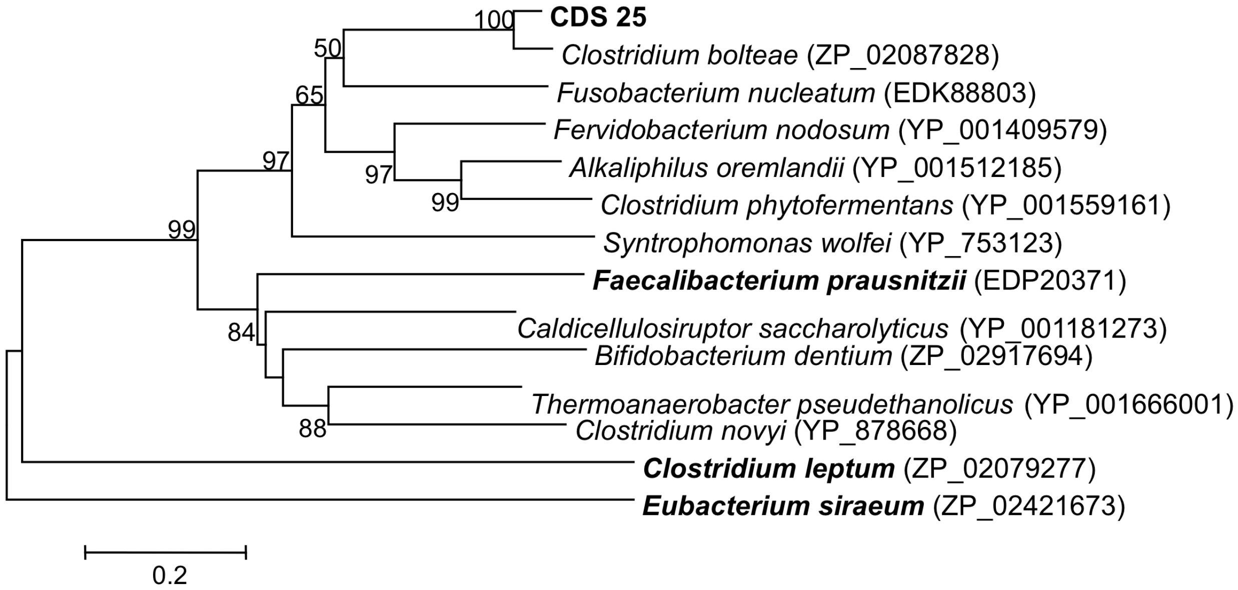

o) CDS 26

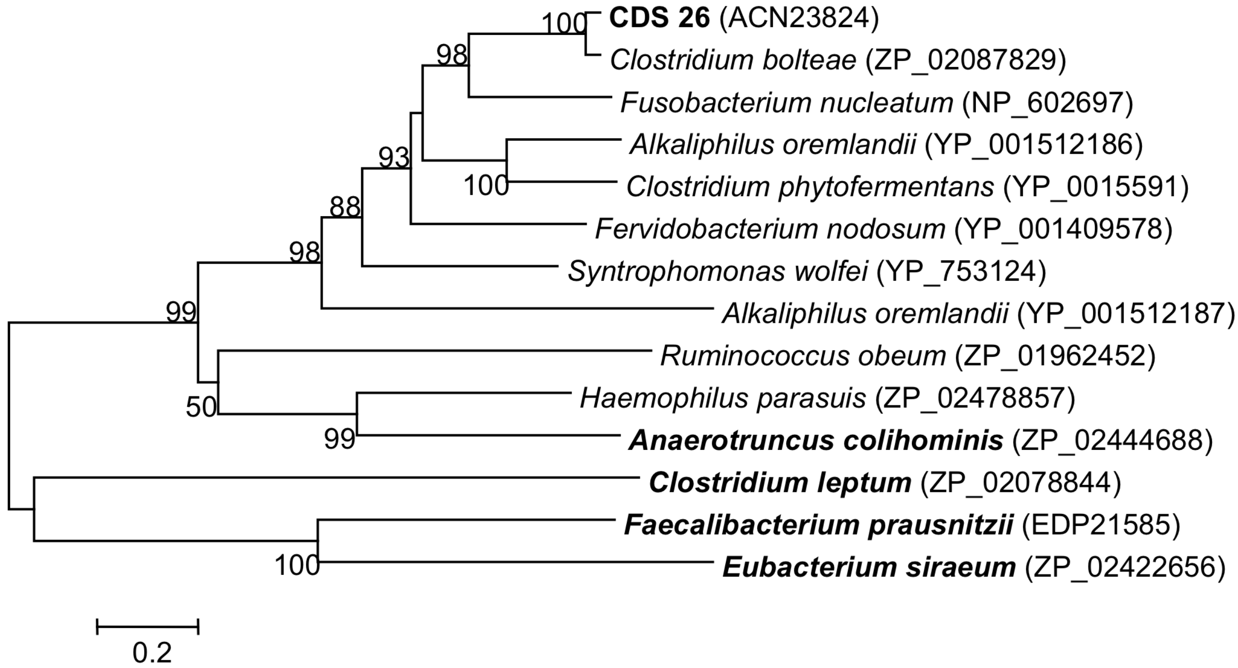

p) CDS 27

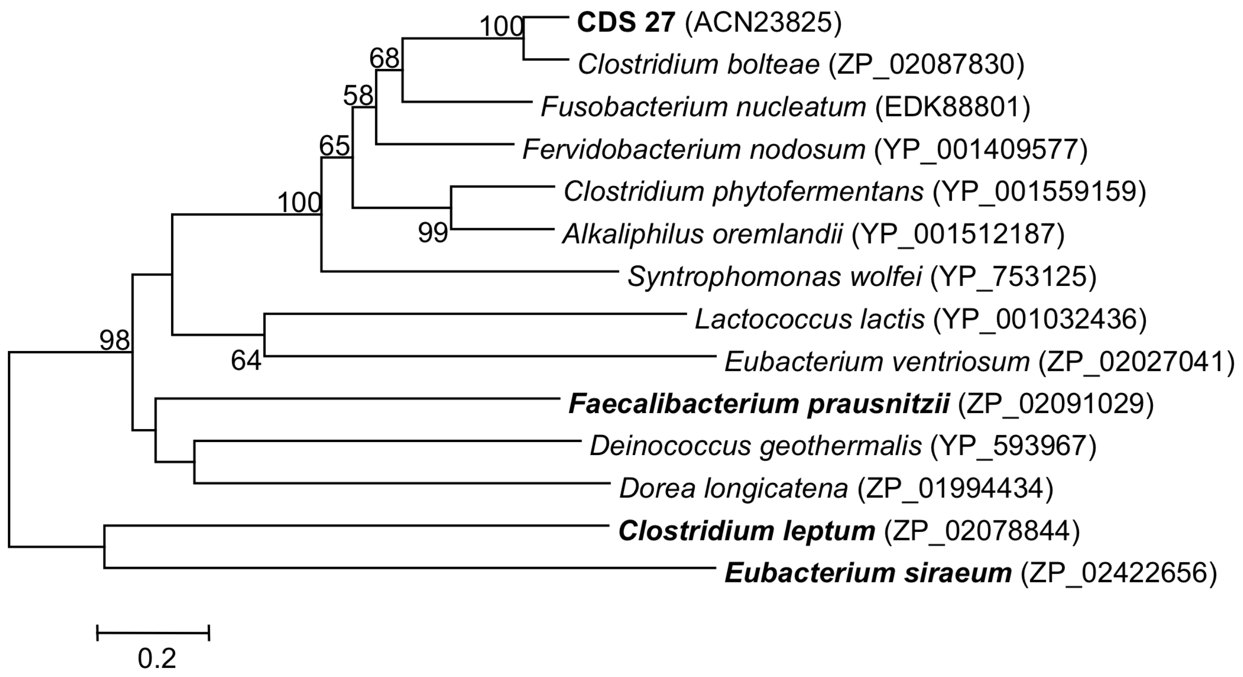

q) CDS 28

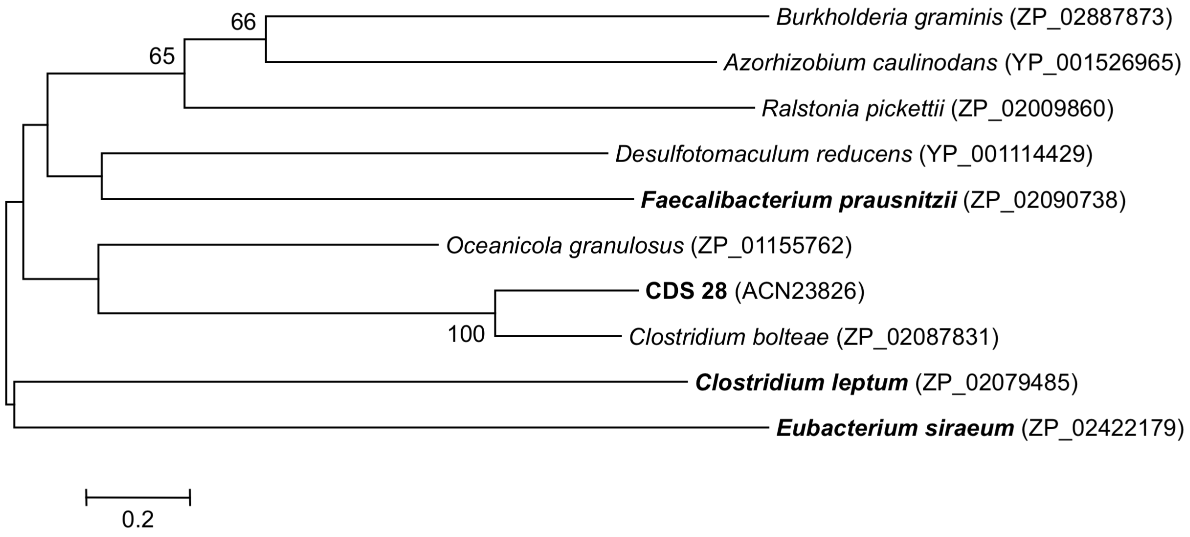

Supplement: Figure S3 — Neighbor-joining phylogenetic trees of amino acid sequences from fosmid 7–25 that were used to assess LGT, as described in the text. Sequences in bold represent those from Clostridium cluster IV. For CDS 18 the conclusion of LGT based upon the neighbor-joining tree differed from that based upon the maximum likelihood tree (as listed in Table 1). Thus for this CDS we also show the maximum likelihood tree. (3.02 MB PDF) [file pone.0010785.s003.pdf]
